# Peptide-Coated Polycaprolactone-Benzalkonium Chloride Nanocapsules for Targeted Drug Delivery to the Pancreatic β-Cell

**DOI:** 10.1021/acsabm.4c00621

**Published:** 2024-09-24

**Authors:** Jillian Collins, Jessie M. Barra, Keifer Holcomb, Andres Ocampo, Ashton Fremin, Austin Kratz, Jubril Akolade, Julianna K. Hays, Ali Shilleh, Amit Sela, David J. Hodson, Johannes Broichhagen, Holger A. Russ, Nikki L. Farnsworth

**Affiliations:** † Department of Chemical and Biological Engineering, Colorado School of Mines, Golden, Colorado 80401, United States; ‡ Depart of Pharmacology and Therapeutics, Diabetes Institute, University of Florida, Gainesville, Florida 32610, United States; § Oxford Centre for Diabetes, Endocrinology and Metabolism (OCDEM), NIHR Oxford Biomedical Research Centre, Churchill Hospital, Radcliffe Department of Medicine, University of Oxford, Oxford OX3 9DU, United Kingdom; ∥ Leibniz-Forschungsinstitut für Molekulare Pharmakologie, Robert-Roessle-Str. 10, Berlin 13125, Germany

**Keywords:** nanomedicine, nanocapsules, polycaprolactone, targeted drug delivery, pancreatic islets, β-cell, diabetes

## Abstract

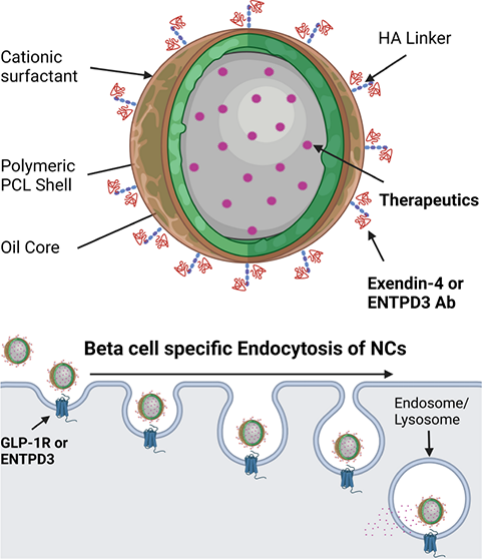

Targeting current therapies to treat or prevent the loss of pancreatic islet β-cells in Type 1 Diabetes (T1D) may provide improved efficacy and reduce off-target effects. Current efforts to target the β-cell are limited by a lack of β-cell-specific targets and the inability to test multiple targeting moieties with the same delivery vehicle. Here, we fabricate a tailorable polycaprolactone nanocapsule (NC) in which multiple different targeting peptides can be interchangeably attached for β-cell-specific delivery. Incorporation of a cationic surfactant in the NC shell allows for the attachment of Exendin-4 and an antibody for ectonucleoside triphosphate diphosphohydrolase 3 (ENTPD3) for β-cell-specific targeting. The average NC size ranges from 250 to 300 nm with a polydispersity index under 0.2. The NCs are nontoxic, stable in media culture, and can be lyophilized and reconstituted. NCs coated with a targeting peptide were taken up by human cadaveric islet β-cells and human stem cell-derived β-like cells (sBC) in vitro with a high level of specificity. Furthermore, NCs successfully delivered both hydrophobic and hydrophilic cargo to human β-cells. Additionally, Exendin-4-coated NCs were stable and targeted the mouse pancreatic islet β-cell in vivo. Overall, our tailorable NCs have the potential to improve cell-targeted drug delivery and can be utilized as a screening platform to test the efficacy of cell-targeting peptides.

## Introduction

Type 1 diabetes (T1D) is a global health problem affecting 8.4 million people worldwide, and the number of diagnosed cases is projected to double by 2040.^1^ T1D is characterized by the targeted destruction of insulin-producing pancreatic islet β-cells.^1^ This leads to impaired glucose homeostasis and a significant decline in quality of life.^2^ Although exogenous insulin injections can restore glucose homeostasis, they do not protect against life-threatening disease complications, are susceptible to dosage errors, and do not stop disease progression.^3^ There are a variety of therapies in development to protect β-cell mass and function as a preventative treatment for T1D.^4^ There is evidence that T1D patients still retain some residual β-cell mass.^4,5^ Therefore, developing pharmacologic approaches to safeguard and promote the proliferation of residual β-cells and preserve graft mass and function post-transplantation could be an alternative T1D therapy. Dual specificity tyrosine-phosphorylation-regulated kinase 1A (DYRK1A) inhibitors, for example, are a class of molecules that can induce the proliferation of human β-cells.^4^ However, DYRK1A inhibitors can also inhibit proliferation at higher doses and are not specifically targeted to the β-cell.^4,6^ Other therapeutics in development include the calcium channel blocker Verapamil, which promotes β-cell function in adults with new-onset T1D; however, targeted delivery of increased concentrations of Verapamil to the β-cell may further improve β-cell function and survival without the off-target effects of hypotension that occur with oral administration.^7^ Therefore, there is a critical need for targeted drug delivery specifically to human β-cells for treating T1D.

Intravenous administration of therapeutics with effects on the pancreatic β-cell can bypass the gastrointestinal tract, requires lower dosage, and can reach the exocrine pancreas via the vascular system.^8,9^ However, intravenous drug delivery strategies face a myriad of biological barriers before reaching their target site and have significant off-target effects. To circumvent these challenges, utilizing nanocarriers for targeted drug delivery has been expanding due to their small size and customizable surface chemistry, allowing the attachment of targeting moieties to improve cell specificity and overcome biological barriers.^10−12^ Nanocapsules (NCs) have demonstrated their ability to efficiently deliver small peptides and other therapeutics directly to the desired cellular targets in a controlled manner.^13,14^ NCs are a type of nanoparticle consisting of an outer biodegradable polymer shell encompassing a liquid or solid core, which have demonstrated longer release of cargo compared to nanospheres.^15,16^ Poly(ε-caprolactone) (PCL) is a hydrophobic semicrystalline polymer extensively used in nanoparticle formulations for drug delivery.^17,18^ In addition to being biocompatible and biodegradable, PCL nanoparticles have exhibited slow and sustained release of a variety of cargo, including small molecules, peptides, and RNA.^19−21^ Furthermore, PCL NCs composed of an oil core encapsulated inside a PCL polymer shell can successfully encapsulate hydrophilic or hydrophobic cargo and deliver therapeutics in a controlled manner.^15,22^ Although nanoparticles provide controlled drug release, cell-specific targeting remains challenging due to a lack of cell-specific surface markers.^12,23,24^ This supports a pressing need for innovative drug delivery platforms that provide a higher throughput screening method for cell specificity with multiple cell surface targets.^11,25^

Nanoparticles targeted to the pancreatic islet β-cell have previously been utilized as a noninvasive strategy for quantifying and visualizing β-cell mass.^26,27^ Notably, iron nanoparticles coated with Exendin-4 (Ex4), an agonist for the glucagon-like peptide 1 receptor (GLP-1R) that is expressed on the surface of β-cells, were successful in targeting the pancreatic islet in vivo.^26,28,29^ Although these magnetic nanoparticles could potentially be used as an early diagnostic tool for diabetes detection,^30,31^ implementing nanoparticles for targeted drug delivery to protect and promote the proliferation of pancreatic β-cells has not been fully investigated. Furthermore, the endocytic pathway of GLP-1R in pancreatic β-cells has been characterized.^32^ However, while GLP-1R has been used in several studies to target the islet β-cell for imaging or delivery of therapeutics, accumulation of Ex4-targeted cargo in off-target tissues such as the spleen, kidney, liver, and lungs has been observed and may be attributed to GLP-1R expression in the lungs and liver.^28,33^ Therefore, to improve targeted delivery to islet β-cells, a tailorable drug delivery vehicle with interchangeable targeting moieties is needed to identify novel peptides with high β-cell specificity.

In this study, we synthesized a novel NC drug delivery platform using PCL NCs coated with the cationic surfactant benzalkonium chloride (BKC)-PCL-NCs. The charged surfactant allows the adherence of different moieties for specific cell targeting. We demonstrated that the NCs can target pancreatic β-cells using two different targeting molecules on the NC surface, highlighting the modular capabilities of our approach. Notably, NCs can encapsulate either hydrophilic or hydrophobic cargo with sustained release for prolonged periods (∼40 days) and are stable under physiological conditions. NCs could be coated with either Ex4 or an antibody for ectonucleoside triphosphate diphosphohydrolase 3 (ENTPD3) for β-cell-specific targeting. NCs selectively target human stem cell-derived β-like cells (sBCs) and human cadaveric islet β-cells in vitro and can deliver therapeutic cargo. Additionally, NCs are stable after tail vein injection into NOD-Scid mice and can selectively target pancreatic β-cells in vivo. The results from this study support proof of the principle of targeted delivery to the human β-cell using our innovative NC design. The ability to selectively target and deliver therapeutic cargo to human pancreatic β-cells via NCs significantly improves the prospect of protecting, proliferating, and regenerating replacement or residual pancreatic β-cells for T1D patients.

## Results and Discussion

### Feasible to Fabricate BKC-PCL-NCs and Encapsulate Either Hydrophobic or Hydrophilic Cargo

**Figure 1:**
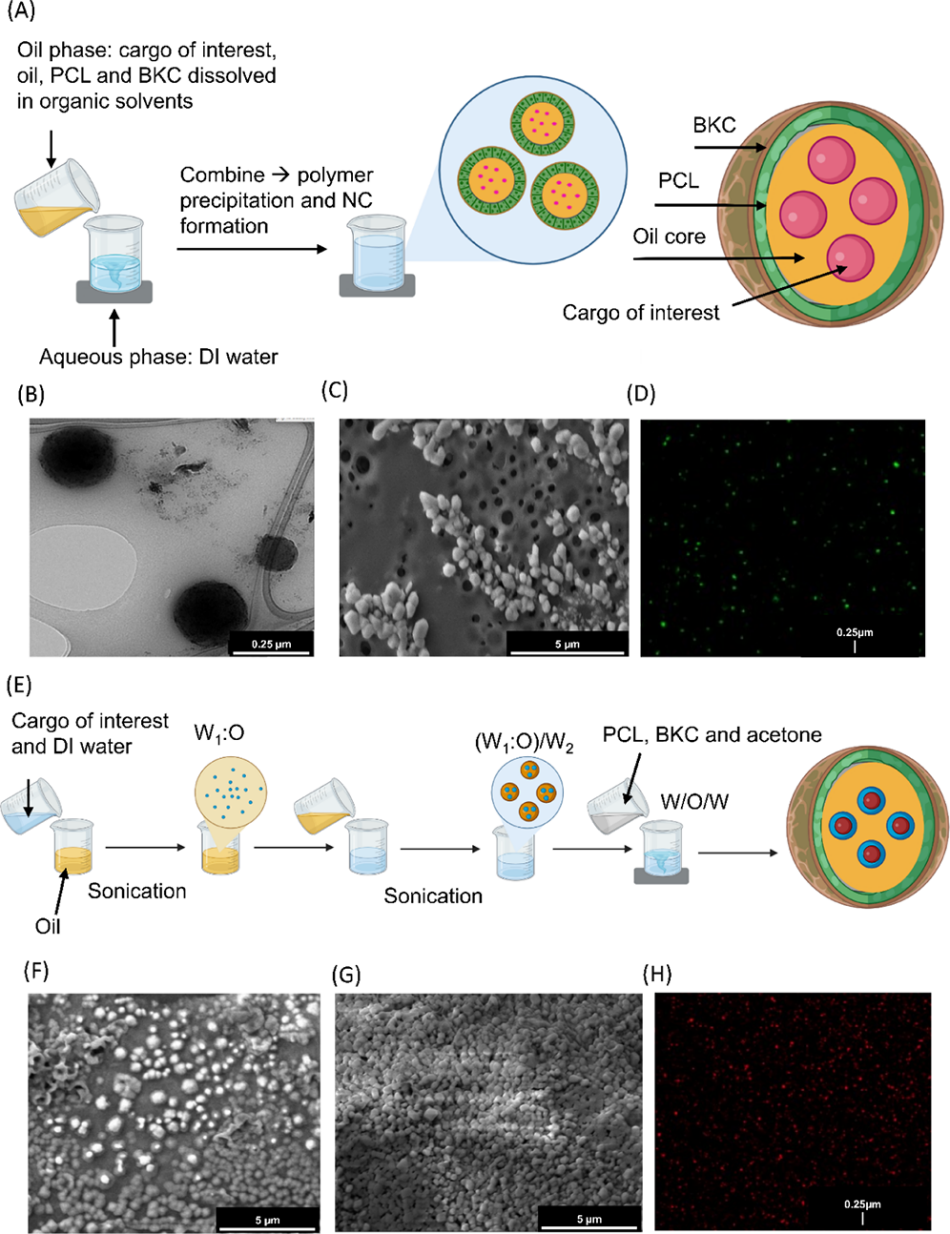
Water -in -oil (W/O) and water -in -oil -in -water (W/O/W) nanocapsule (NC) synthesis. (A) Schematic outlining water-in-oil (W/O) synthesis of NCs where the cargo of interest is stored in an oil core surrounded by a polycaprolactone (PCL) polymer shell coated with the cationic surfactant benzalkonium chloride (BKC), created in BioRender. The oil phase was combined with the aqueous phase under vigorous stirring for NC precipitation. Representative transmission electron microscopy (TEM) (B) and scanning electron microscopy (SEM) (C) images of blank (no cargo) W/O NCs. (D) Representative confocal image of W/O NCs loaded with BODIPY. (E) Schematic outlining water-in-oil-in-water (W/O/W) synthesis of NCs created in BioRender. The primary emulsion (W1:O) consists of the cargo of interest dissolved in DI water combined with the oil phase under sonication. The primary emulsion was combined with a secondary aqueous phase under sonication (W1:O)/W2. This was combined with an organic solvent containing the polymer and surfactant under rigorous stirring. Representative SEM images of (F) uncoated and (G) BSA-coated blank W/O/W NCs. (H) Representative confocal image of W/O/W NCs loaded with Rh123. In images B–D and F–H, the scale bar was added in postprocessing to enlarge the text size using ImageJ.

Coated PCL nanoparticles have been utilized in a myriad of drug delivery applications.^15,34,35^ It has been previously demonstrated that BKC can integrate into the PCL polymer framework via electrostatic interactions to form solid nanospheres, but this has not been demonstrated in a nanocapsule (NC).^35^ Furthermore, BKC’s cationic nature allows the opportunity to adhere targeting peptides to the outside of such particles. BKC is an FDA-approved preservative already used for ophthalmic applications and is capable of coating nanoparticles for clinical applications.^35−37^ Therefore, we modified existing NP protocols and optimized them to achieve stable NCs with BKC incorporated into the PCL shell (BKC-PCL NCs).^15,35^ For hydrophobic cargo, NCs were synthesized via a water-in-oil (W/O) emulsion (Figure 1A). Briefly, the oil phase containing the cargo of interest was combined with an organic solution containing PCL and BKC, which was then combined with the aqueous phase under rigorous stirring. BKC concentrations, oil core volumes, and rates of stirring were altered to determine the optimal NC formulation (Table 1A–C). BKC concentration had the largest standard variation of the mean (StdEM) on both NC size (StdEM ± 640 nm) and polydispersity index (PDI) (StdEM ± 0.12), followed by changes in oil volumes (StdEM ± 16 for NC size and StdEM ± 0.03 for PDI) and then stirring speeds (StdEM ± 12 for NC size and StdEM ± 0.02 for PDI) (Table 1A–C). This is consistent with previous studies that showed similar changes in relative nanoparticle size with similar changes in stir speed and oil volume.^38−40^ Out of all combinations tested, the synthesis parameters chosen for the rest of the study for the W/O emulsion consisted of the following: BKC (20wt/wt % PCL), 60 μL oil, and 850 rpm stirring speed, which yielded the smallest average NC diameter of 271 ± 7 nm with an average PDI of 0.06 ± 0.03, respectively (Table 1A). The spherical shape diameter and relative monodispersibility were confirmed by SEM and TEM (Figure 1B,C). Furthermore, the successful encapsulation of BODIPY, a hydrophobic fluorescent cargo, was confirmed via confocal microscopy (Figure 1D).

**Table 1:**
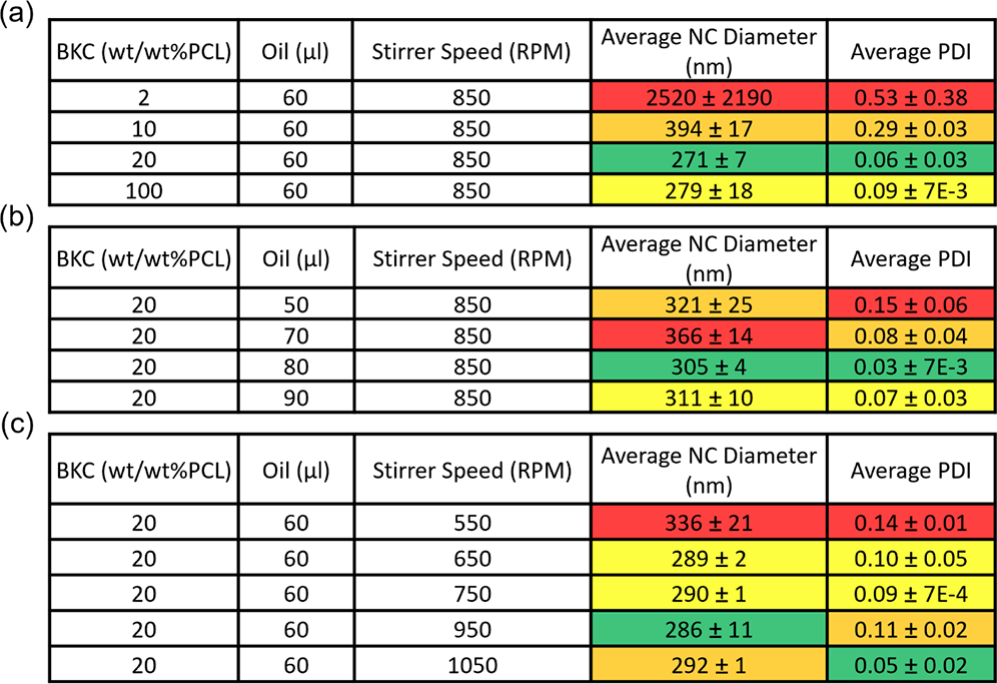
Evaluation of W/O NC Size and Polydispersity Index (PDI) with Changing Synthesis Parameters, Including (a) BKC Concentrations, (b) Oil Core Volumes, and (c) Rates of Stirring (*n* = 3)^a^

a Values are color-schemed as follows: green, the lowest average values; yellow, the second or third lowest values if there are more than four values for NC size and PDI; orange, the second highest value for NC size and PDI; red, the highest values.

For hydrophilic cargo encapsulation, NCs were synthesized via a water-in-oil-in-water (W/O/W) emulsion (Figure 1E). For optimization of this fabrication method, both W:O and (W_1_:O)/W_2_ ratios were altered for the W/O/W NCs (Table 2). These factors were varied to obtain the maximum NC yield while maintaining the desired NC size. NCs formulated with a 25:75 W_1_:O and 10:90 (W_1_:O)/W_2_ had a significantly higher NC diameter than the other two treatments with a 10:90 (W_1_:O)/W_2_ phase (*p* = 0.03 for 40:60 W_1_:O and *p* = 0.02 for 50:50 W_1_:O, Table 2). This supports the idea that varying the ratios of each phase can influence NC size. Like the W/O NCs, the average diameter and relative monodispersibility of coated and uncoated W/O/W NCs were confirmed by SEM (Figure 1F,G). Successful encapsulation of rhodamine 123 (Rh123), a hydrophilic fluorescent cargo, for W/O/W NCs (Figure 1H) was also confirmed by confocal microscopy.

**Table 2:**
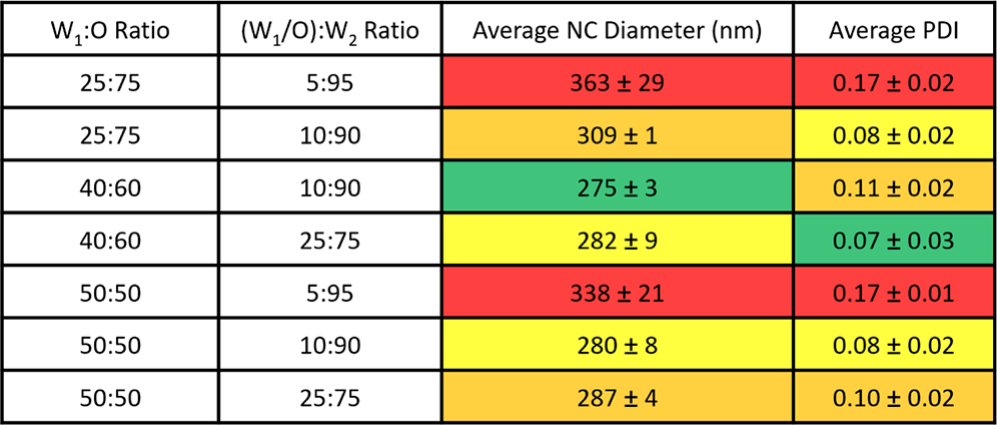
Assessment of Different W/O Ratios and W/O/W Ratios on W/O/W NC Size and Polydispersity Index (PDI) (*n* = 3)^a^

a All ratios are reported as volumes in jU. Values are color-schemed as follows: green, lowest average value; yellow, values within 10 nm of the lowest value for NC size and 0.01 for PDI; orange, values within 50 nm of the lowest value for NC size or 0.05 for PDI; red, values over 50 nm of the lowest value for NC size or over 0.1 for PDI.

**Figure 2:**
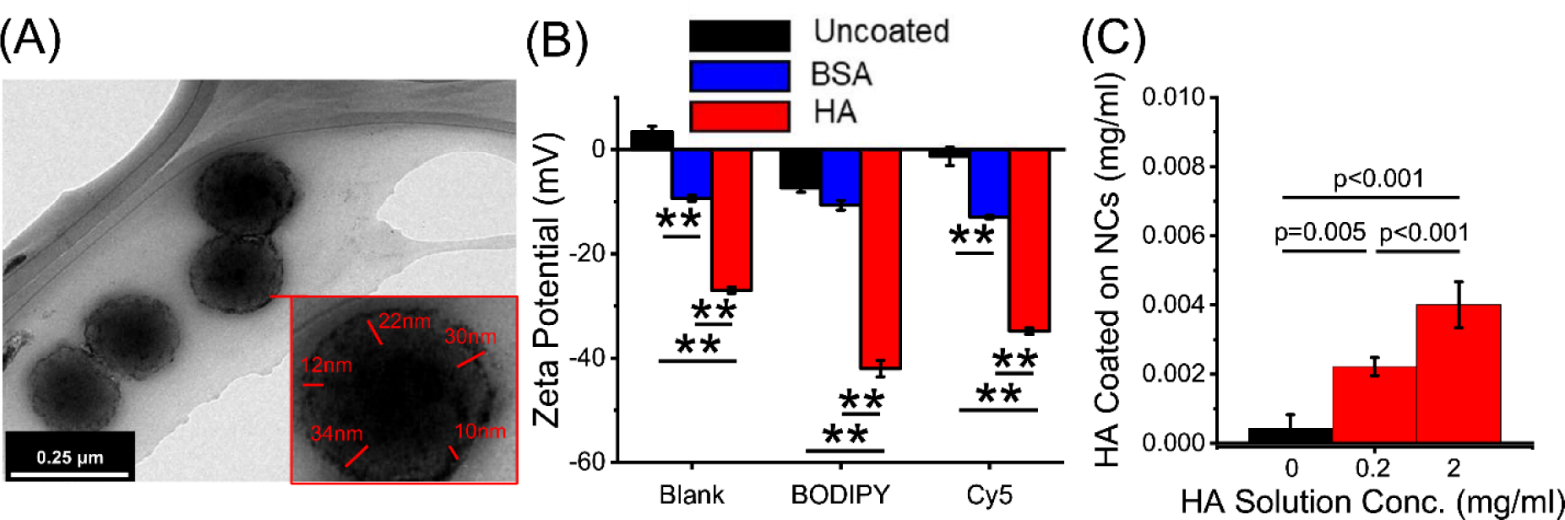
Various coatings can be applied to the outside of the NCs. (A) TEM image of BSA (5 mg/mL)-coated W/O NCs with a zoomed-in NC highlighting the width of the BSA coating as measured in ImageJ. (B) Zeta potential of W/O NCs uncoated, BSA (5 mg/mL), and HA (0.2 mg/mL) coated and either unloaded (blank) or loaded with BODIPY or Cy5 (*n* = 3). (C) Concentration of HA in NC emulsions after coating with 0, 0.2, or 2 mg/mL HA and washing for 10 000 rpm for 10 min and resuspending capsules in HA-free buffer. **p < 0.001 and *p* = 0.005 were considered significant as determined via ANOVA with Tukey’s post hoc analysis.

Cargo were tested with varying charge, size, and hydrophobicity to assess if these parameters influenced NC stability, size, and encapsulation efficiency (EE). There was less than a 20 nm increase in the NC diameter for the W/O NC diameter when encapsulating either BODIPY or Cy5, while still maintaining a homogeneous size distribution (Figure 2A, Table 3A), which is consistent with other studies.^41,42^ However, the Rh123-loaded W/O/W NCs had a 63 nm diameter increase as well as a slight increase in the PDI (Table 3B). The bulky structure of Rh123^43^ could be attributed to the larger increase in NC size, as another nanoparticle study showed about a 50 nm increase with Rh123 NPs compared to the unloaded control.^44^ These data suggest that the NC diameter can be changed based on the size of the cargo being encapsulated. The EE of the BODIPY-encapsulated W/O NCs, as determined using eq 1, was 68 ± 1.3% and 47 ± 0.7% for Rh123-encapsulated W/O/W NCs (Table 3C). The EE for BODIPY is similar to other NP fromulations^45^; however, Rh123 is much lower.^46^ The lower EE could be attributed to losing some of the solution during the transfer steps, or the Rh123:PCL concentration was too high and there was not enough PCL to encapsulate all the Rh123. Overall, these data support that our NCs can be designed to encapsulate either hydrophobic or hydrophilic cargo.

**Table 3:** Influence of Different Encapsulated Cargos on the Size and Polydispersity Index (PDI) of (a) W/O NCS and (b) W/O/W NCs, and (c) Percent of Cargo That Was Successfully Encapsulated (Encapsulation Efficiency) for BODIPY (Hydrophobic) in W/O and Rhodamine (Hydrophilic) for W/O/W NCs (*n* = 3–5)^a^

Encapsulated Cargo	Average NC Diameter (nm)	Average PDI
(a)		
BODIPY	278 ±11	0.08 ± 0.04
Cy5	286 ± 30	0.08 ± 0.01

Encapsulated Cargo	Average NC Diameter (nm)	Average PDI
(b)		
rhodamine	338 ± 21	0.17 ± 0.02
pentamidine	282 ± 14	0.11 + 0.01

(c)			
Encapsulated Cargo	Fluorophore Loading	% Encapsulation Efficiency	*R*^2^
BODIPY (W/O)	10 μg	68 ± 1.3	0.99
rhodamine (W/O/W)	10 μg	47 ± 0.7	0.99

a The coefficient of determination (*R*^2^) illustrates the fit of the standard curve to the actual encapsulation efficiency data.

### Anionic Coatings Can Adhere to NCs and Improve NC Stability

We next tested whether BKC-PCL NCs could be coated with anionic coatings to improve NC stability and enhance the conjugation of different moieties for targeted delivery. Bovine serum albumin (BSA) and hyaluronic acid (HA) were chosen as they have different anionic strengths and can be used for in vitro or in vivo work. NCs were coated either with BSA or HA to determine if anionic compounds could adhere to the BKC/PCL framework.^35^ Compared to the average uncoated W/O NC diameter of 271 ± 7 nm, HA-coated NCs had an average diameter of 293 ± 4 nm and BSA-coated NCs had an average diameter of 295 ± 1 nm (Table 4). This equates to an average diameter increase of 22 nm for HA-coated W/O NCs and 24 nm for BSA-coated W/O NCs, supporting that anionic coatings do not greatly alter NC size. Scanning electron microscopy (SEM) and transmission electron microscopy (TEM) images both support the dynamic light scattering (DLS) measurements (Figures 1B,F,G and 2A). Furthermore, measurements were taken from the TEM images between the lighter shell (BSA coating) and darker core (NC) from the longest and shortest widths, which averaged between 20 and 30 nm, supporting the increase in NC size displayed by DLS (Figure 2A and Table 4). All PDIs were under 0.1, and there was only a 0.01 difference between the coated and uncoated NCs, demonstrating that all formulations were monodispersed. The ability to adhere anionic coatings to the NCs allows the potential to attach various moieties to improve target specificity.

**Table 4:** Change in NC Size and Size Distribution of Uncoated and Coated W/O Synthesized NCs (*n* = 3)

Coating	Average NC Diameter (nm)	Average PDI
none	271 ±7	0.06 ±0.03
HA (0.2 mg/mL)	293 + 4	0.05 ±0.02
BSA (5 mg/mL)	295 ± 1	0.05 ±0.01

**Figure 3:**
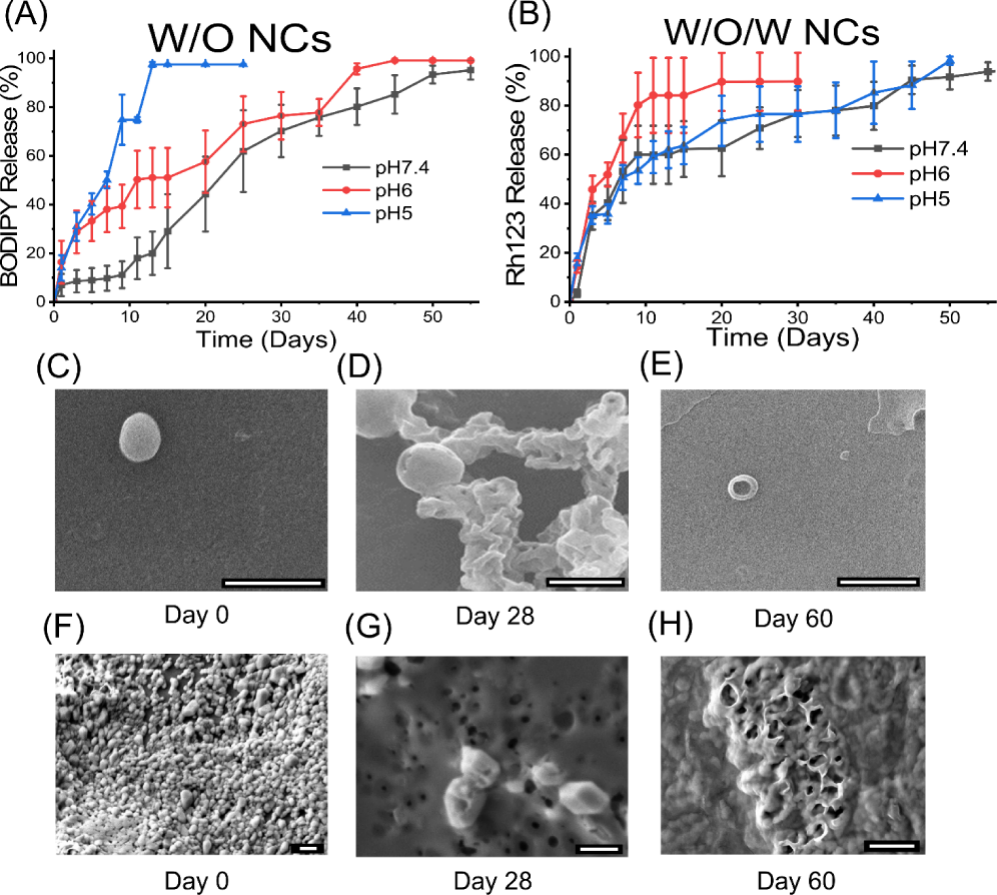
NCs can degrade and release cargos of interest at varying pHs. (A) In vitro release profiles of W/O NCs loaded with BODIPY and (B) in vitro release profiles of W/O/W NCs loaded with Rh123 in 1× PBS at pH 7.4, 6, and 5 (*n* = 3–5). Release data are presented as the average value ± standard error of the mean. All release data were normalized by subtracting the fluorescent value at time 0 and then dividing by the maximum fluorescence value (100% release). Representative SEM images of W/O NC degradation (C–E) and W/O/W NC degradation (F–H) on Days 0, 28, and 60 in 1× PBS at pH 7.4. Scale bars are 1 μm and were added in image postprocessing using ImageJ.

W/O NC surface charge, as measured by the zeta potential, was utilized to confirm if BSA and HA adhered to the outside of the NCs and to assess the stability of the coating. Furthermore, it was utilized to assess if encapsulated cargo, BODIPY (anionic) and Cy5 (cationic), significantly altered the NC charge (Figure 2B). Although Cy5-loaded NCs did have a slight increase in zeta potential, it did not significantly alter the NC charge compared to the blank W/O NCs (Figure 2B). This was consistent with a previous study where Cy5 itself was not a significant contributor to changes in zeta potential.^41^ Although the zeta potential of the uncoated BODIPY NCs was consistent with previous studies,^42^ there was a significant negative charge compared to both the uncoated blank (*p* < 0.001) and uncoated Cy5 W/O NCs (*p* = 0.013, Figure 3B). When comparing the uncoated to both HA- and BSA-coated W/O NCs, all conditions tested, except the blank BSA-coated and BODIPY-loaded BSA-coated W/O NCs, had a significant negative charge (*p* < 0.001) ranging from −9 to −12 mV for BSA and −27 to −42 mV for HA (Figure 3B). To further confirm that the NC coating was stably adhered to the surface of the NCs, we utilized a fluorescently tagged HA to confirm that HA remains adhered to the NCs after washing with centrifugation (Figure 2C). HA was detected on NCs after washing with centrifugation at 10 000 rpm for 10 min and resuspending NCs in PBS. Additionally, increasing the HA solution concentration increased the amount of HA coated on the NCs (Figure S1A). In addition, when comparing different BSA concentrations, 10 mg/mL of BSA-coated BODIPY-encapsulated W/O NCs had a significant negative charge compared to other concentrations (*p* < 0.001, Figure S1B). These results are critical as charge can influence important factors such as cytotoxicity, cell permeability, and efficacy.^47−49^ The ability to tightly control surface charge allows the conjugation of ligands, peptides, antibodies, etc., for improved cell specificity, making them a valuable drug delivery vehicle as well as a screening tool for cell-specific moieties for biological applications.

### NCs Can Be Freeze-Dried and are Stable in Culture Media

Freeze-drying is a method used to preserve NP integrity for extended periods of time and will allow the translation of this technology into a clinical setting where storage for prolonged periods of time will be required.^50^ Therefore, we freeze-dried NCs and assessed their stability over varying amounts of time. Mannitol was chosen as the cryoprotectant to help prevent aggregation and maintain NC stability.^50^ Various mannitol concentrations were tested to determine the optimal concentration (Figure S2A). There were no significant increases in NC size before and after freeze-drying and reconstituting NCs in DI water between the different mannitol concentrations (Figure S2A). Untreated NCs without cryoprotectants did not survive the freeze-drying process, demonstrating that a cryoprotectant is required for freeze-drying (Figure S2A). SEM images were taken before (Figure S2B) and after (Figure S2C) freeze-drying to confirm DLS measurements. It is important to note that the NC yield decreased after reconstitution, which may be attributed to the mannitol crystallizing and imposing mechanical forces, either breaking the NCs or forming aggregates.^50^ Future work will include investigating other cryo- or lyoprotectants as well as steric stabilizers, which can improve NC stability.^50^

Lastly, the stability of uncoated, HA-coated, and BSA-coated W/O NCs was tested in Dulbecco’s modified Eagle’s medium (DMEM) culture media in vitro, as well as after being extracted and passed through a 29G 1 cm^3^ insulin syringe. There was no significant difference in the NC size or PDI after passing NCs through a 29G syringe, as well as being incubated in DMEM for 1 and 24 h either at 37 °C or room temperature (Figure S3A–C). All PDIs for all conditions were under 0.12. Overall, the characterization data demonstrate the feasibility, stability, and interchangeable capabilities for both charge and encapsulated cargo of the NCs. This supports the stability of NCs in vitro and the delivery of NCs via IV injection through a 29G syringe for in vivo experiments.

### In Vitro Cargo Release of W/O and W/O/W NCs

To determine the release profile of cargo from BKC-PCL NCs, BODIPY (W/O) and Rh123 (W/O/W)-loaded NCs were placed in 1× PBS reservoirs at varying pHs, and release rates were measured by the fluorescence intensity of the solution using eqs 2 and 3. For the BODIPY-loaded W/O NCs, the total average release time was between 45 and 50 days when incubated in PBS at pH 7.4 (Figure 3A). There was no significant difference at any time point between pH 7.4 and pH 6. For pH 5 and pH 6 NCs, both had a similar increase in the release rate in the first 24 h (Figure 3A). Although pH 6 and pH 5 NCs have a higher initial release rate compared to pH 7.4, there was only a significant difference in release between NCs incubating in pH 7.4 versus pH 5 on Day 6 (*p* = 0.004) and Day 7 (*p* = 0.004) in the first week (Figure 3A). NCs at pH 5 almost have 100% of the cargo released by the end of the second week (Figure 3A). Furthermore, pH 5 had a significantly faster release profile compared to pH 7.4 every day during the second week (*p* = 0.12, *p* < 0.001, *p* = 0.001, *p* = 0.002, *p* = 0.004, *p* < 0.001, and *p* = 0.002) and only on the 13th (*p* = 0.04) and 14th day (*p* = 0.04) compared to pH 6. There was no significant change at any time point in the release profiles for Rh123-loaded W/O/W NCs (Figure 3B); however, the release profile for W/O/W NCs was faster over the first week and then more gradual over progressive weeks compared to the W/O NCs. SEM images taken on Days 0, 30, and 60 confirmed the degradation morphology of both the W/O and W/O/W NCs (Figure 3C–H). By Day 30, pores began to appear on the NC surface (Figure 3D,G), supporting the release of fluorescent cargo, as illustrated by the release studies (Figure 3A,B). By Day 60, it appears that all cargo has been released (Figure 3A,B) and only a partial PCL shell remains (Figure 3E,H).

These results support that pH influences the degradation and, in turn, the release profiles of the W/O NCs. The faster degradation rate at lower pH is likely caused by increased hydrolytic degradation by random chain fission, as this has been demonstrated as a major contributor in PCL degradation.^18^ Understanding this pH-dependent mechanism is imperative because if the NCs are successfully endocytosed, they may be captured by cellular organelles such as lysosomes or endosomes, which have lower pH values (4–6) leading to faster drug release times.^51^

### NCs are Nontoxic and Can Selectively Target Insulin-Producing sBCs and Human Islets In Vitro

**Figure 4:**
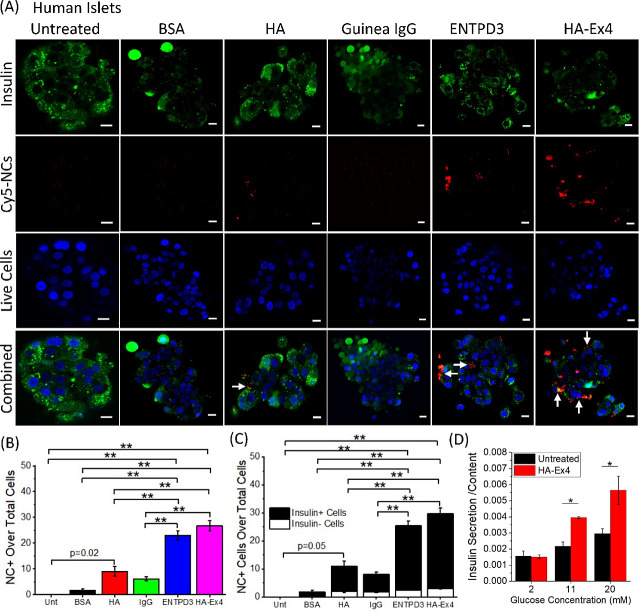
NCs specifically target insulin producing sBCs and human islet cells in vitro. (A) Representative confocal images showing insulin (green), live cell nuclei (blue), and Cy5 NCs (red) with various NC coatings. White arrows indicate NC+ and insulin+ cells. The scale bar is 10 μm for all images. (B) Quantification of NC uptake with intact human islets. NCs were uncoated or coated with BSA (5 mg/mL), HA (0.2 mg/mL), guinea pig IgG (1 μL/mL), ENTPD3 (1 μL/mL), or HA-Ex4 (0.2 mg/mL) and cultured with human islets for 24h (*n* = 2–3). (C) Quantification of insulin– NC+ and insulin+ NC+ cells with intact human islets for each NC treatment (*n* = 2–3). (D) Analysis of glucose-stimulated insulin secretion in isolated mouse islets at 2, 11, or 20 mM glucose, either untreated or treated with 0.43 ng/mL of HA-Ex4 conjugate (∼10 nM Ex4) for 1 h during the assay (*n* = 2–3). **p < 0.001, *p < 0.01, and *p* < 0.05 were considered significant as determined via ANOVA with Tukey’s post hoc analysis.

The pancreatic islet is a complex microorgan comprised of multiple hormone-secreting cells, the bulk of which are insulin-producing β-cells. GLP-1R is expressed on the outside of pancreatic β-cells and plays an important role in maintaining glucose homeostasis.^32^ GLP-1 agonist Ex4 has been utilized extensively for quantifying and visualizing β-cell mass as well as for diabetes therapies.^26,28^ ENTPD3 has recently been shown to be a marker for mature human and stem cell-derived β-like cells; however, it has not been tested as a target for β-cell-specific drug delivery.^52,53^ To assess the NC ability to target β-cells *in vitro*, Cy5-encapsulated NCs were cultured with human islets from 2 to 3 independent donors for 24 h either uncoated or coated with BSA (5 mg/mL), HA (0.2 mg/mL), guinea pig IgG (1 μL/mL), guinea pig ENTPD3 antibody (1 μL/mL), or HA-Ex4 (0.2 mg/mL). The islets were stained with FluoZin, a fluorescent zinc sensor commonly used as a marker for insulin-containing β-cells, and NucBlue (live cell nuclei) and imaged to quantify Cy5 positive (Cy5+ or NC+) and insulin positive (insulin+) cells (Figures 4A and S4A).^54^ Live cell imaging was utilized in lieu of fixed cells to quantify NC uptake, as the fixation process would quench the fluorescence of Cy5 in our NCs. After 24 h, both the ENTPD3 and HA-Ex4 coated treatments had significantly higher NC+ cells compared to the other control NC treatments (Figure 4B). Furthermore, there were ∼6% and 10% increase of Cy5+ in insulin-expressing cells for HA-Ex4 and ENTPD3-coated NCs, respectively, after 48 h incubation compared to 24 h (Figures 4B and S4B). To determine if the NCs were in fact targeting our cells of interest, in this case human β-cells, the NC+ cells were differentiated between insulin+ and insulin– (Figures 4C and S4C). The insulin+ and Cy5+ cells for the ENTPD3 and HAEx4 coated treatments were significantly higher (*p* < 0.001) than the other NC treatments at both 24 and 48 h (Figures 4C and S4C). There were no significant changes observed between all insulin values for all treatments at both 24 and 48 h (Figures 4C and S4C). For the 48 h time point, there was an ∼11% increase in the insulin + NC + cells for the ENTPD3 coated and ∼7% increase with the HA-Ex4 coated (Figures 4C and S4C). There were no significant changes observed between all insulin values for all treatments (Figures 4C and S4C), suggesting that NCs coated with Exendin-4 or ENTPD3 antibody are highly human β-cell specific. Finally, to confirm the bioactivity of HA-Ex4 conjugates coated on the NCs and further support that NC uptake is driven by HA-Ex4 binding to the GLP-1R on β-cells, glucose-stimulated insulin secretion (GSIS) was analyzed from isolated C57Bl/6 mouse islets either untreated or treated with 0.43 ng/mL HA-Ex4 (∼10 nM Ex4 equiv.) during culture in 2 mM, 11 mM, or 20 mM glucose. At both 11 mM and 20 mM glucose, treatment with HA-Ex4 significantly enhanced GSIS (*p* = 0.019 and *p* = 0.002, Figure 4D). This is in line with our previous experiments showing augmentation of GSIS with 10 nM Ex4 in mouse islets and supports that Ex4 maintains its bioactivity after conjugation to HA.^55^

**Figure 5:**
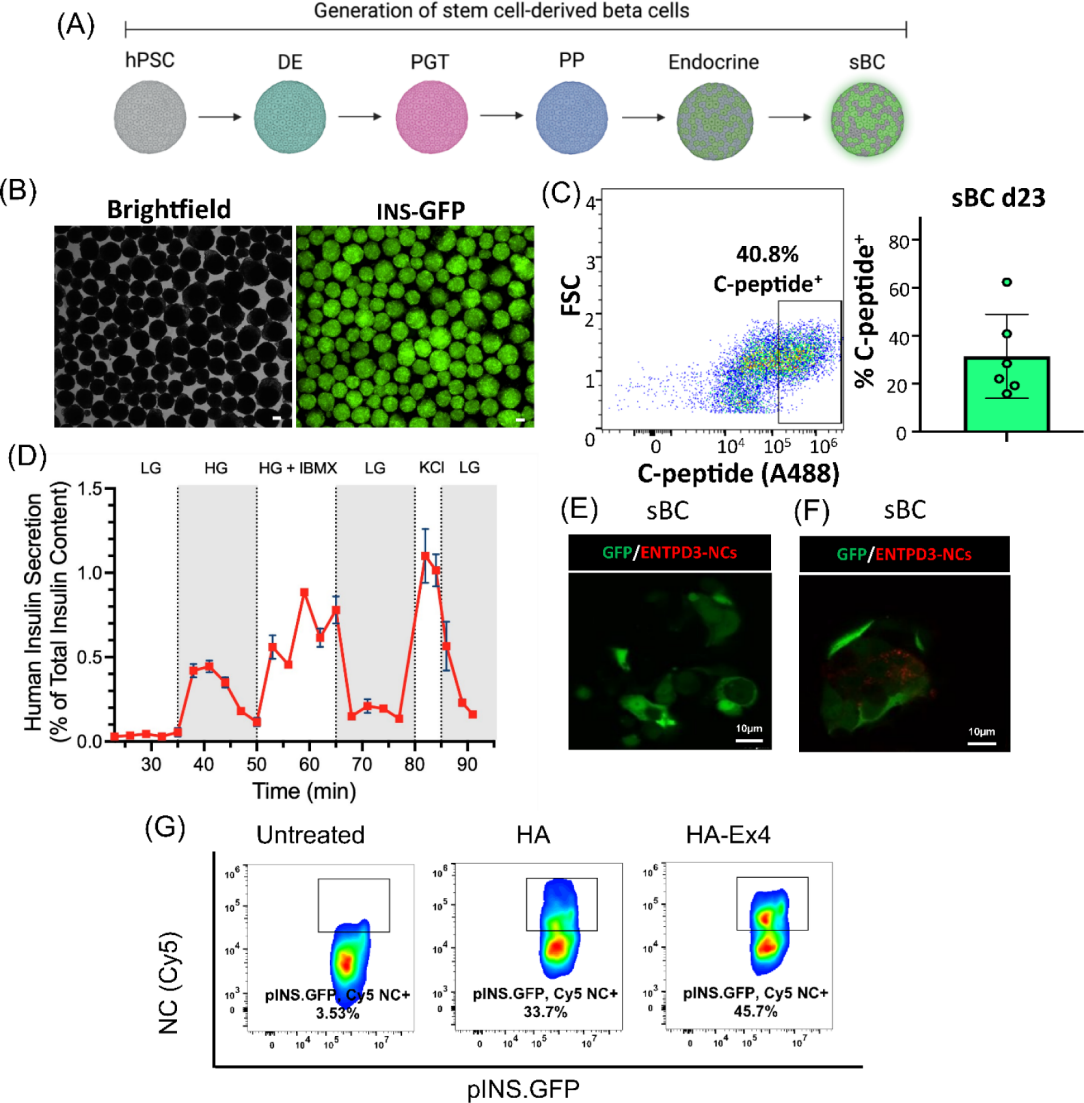
Generation of functional stem cell-derived β-like cells (sBCs). (A) Differentiation schematic to make sBC from human pluripotent stem cells. hPSC – human pluripotent stem cell; DE – definitive endoderm; PGT – pancreatic gut tube; PP – pancreatic progenitor. (B) Representative live bright-field and fluorescent pINS.GFP images of sBC on Day 23 of the differentiation protocol. The scale bar is 100 μm. (C) Representative and summary graph of flow-based quantification of the percentage of C-peptide expressing cells (surrogate for endogenous insulin production), *n* = 6 independent differentiation experiments. (D) Dynamic glucose-stimulated insulin secretion analysis, presented as a percentage of insulin content, of Day 23 sBC. *n* = 2 independent wells from one differentiation experiment. LG – low glucose (2 mM), HG – high glucose (20 mM), IBMX – 3-isobutyl-1-methylxanthine (50 μM), KCl – potassium chloride (30 mM). Representative confocal image of (E) untreated sBCs and (F) Cy5-loaded NCs with sBCs at 24 h (*n* = 1). (G) Flow cytometry quantification of Cy5 signals in live dispersed sBC cocultured with either HA or HA-Ex4 Cy5-loaded NCs at 24 h. Results were pregated on pINS.GFP+ cells (*n* = 1).

Since human islets exhibit considerable variability between isolated preparations, we took advantage of a complementary source of functional human beta cells generated via direct differentiation from pluripotent stem cells as a reproducible and widely used human β-cell model system.^56,57^ We generated sBC from the embryonic human stem cell line Mel1^INS-GFP^ (Figure 5A). These cells have the benefit of a GFP reporter driven by the endogenous insulin promoter, allowing easy visualization of the insulin-producing sBCs (Figure 5B). To characterize the differentiation efficiency of sBC, Day 23 clusters were dissociated, fixed, and stained with a C-peptide (a byproduct that serves as a surrogate marker for endogenous insulin synthesis) antibody labeled with AlexaFluor 488, and analyzed via flow cytometry (Figure 5C). On average, ∼30% of cells were C-peptide positive over six independent differentiations, consistent with previously published results (Figure 5C).^58^ Additionally, analysis of sBC function by dynamic GSIS showed robust insulin secretion in response to high (20 mM) glucose, enhanced insulin secretion with the addition of 50 μM IBMX, and maximal insulin secretion after stimulation with 30 mM KCl (Figure 5D). This demonstrates the functional maturity of differentiated sBC and a dynamic response to glucose challenge that matches previously published results.^59,60^ To determine if the NCs could specifically target insulin-producing sBCs, we cocultured sBC clusters for 24 h with uncoated or NCs coated with ENTPD3 antibody (1 μL/mL) or HA-Ex4 (0.2 mg/mL). Live cell imaging revealed Cy5 + sBC cells, identified by GFP expression, only in ENTPD3-coated NC cultures, further providing evidence for human β-cell-specific targeting using NCs (Figure 5E,F). To confirm these results, we also performed flow cytometry on sBC cocultured with either HA or HA-Ex4 Cy5 NCs for 24h. Assessing our β-cell fraction using the pINS·GFP reporter, we observed that the addition of Ex4 on the surface of our NCs increased the percentage of sBC that took up Cy5-labeled NCs from 33.7% with HA-only-coated NC controls to 45.7% with HA-Ex4-coated NCs (Figure 5G). Despite the significant levels of nonspecific NC uptake with HA-coating, the addition of Ex4 targeting improved uptake by 12% specifically in insulin-expressing cells. Expression of the hyaluronan receptor on many cell types facilitates endocytosis of HA from the extracellular matrix.^61^ While it is unknown if sBC and human islets express this receptor, this may account for the significant nonspecific uptake of HA-coated NCs compared to the IgG-coated capsules in Figures 4 and 5. Overall, our results strongly support that NCs coated with the targeting peptide can be endocytosed by human β-cells with a high level of specificity.

**Figure 6:**
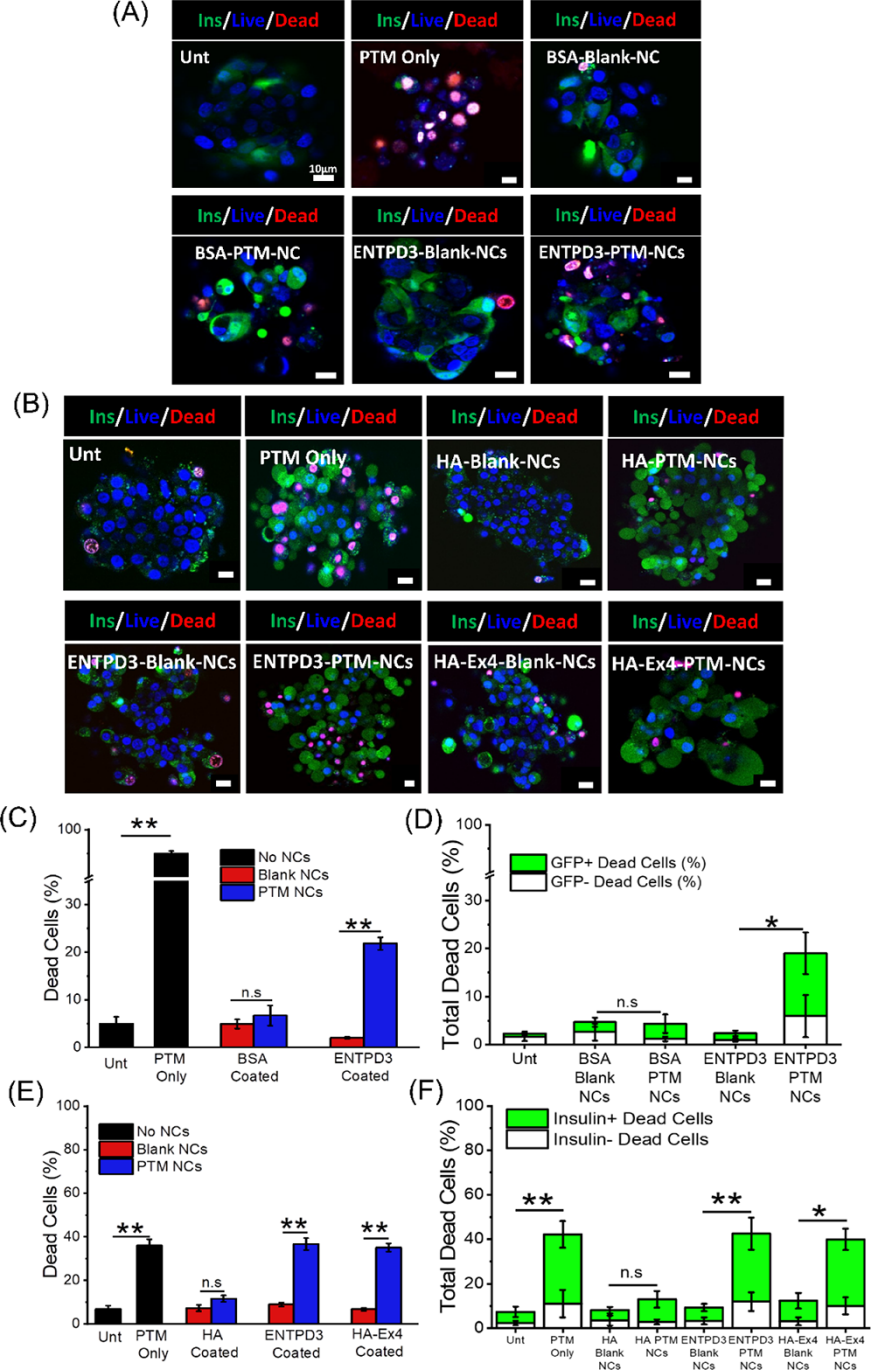
NCs can target, deliver, and release cargo of interest to insulin -producing sBCs and human islets in vitro. (A) Representative confocal images of insulin+ (green), live (NucBlue, blue), and dead (PI, red) sBCs either untreated or treated with pentamidine (PTM) only, blank BSA-coated NCs, PTM-loaded BSA-coated NCs, blank ENTPD3 antibody-coated NCs, or PTM-loaded ENTPD3-coated NCs. (B) Representative confocal images of human islets either untreated or treated with PTM only, blank HA-coated NCs, PTM-loaded HA-coated NCs, blank ENTPD3 antibody-coated NCs, PTM-loaded ENTPD3-coated NCs, blank HA-Ex4-coated NCs, or PTM-loaded HA-Ex4-coated NCs at 24 h. (C) Percentage of total dead sBCs for each NC treatment at 24 h and a breakdown of insulin+ versus insulin– dead cells (D) (*n* = 4–6). (E) Percentage of total dead human islets for each NC treatment at 24 h and a breakdown of insulin+ versus insulin– dead cells (F) (*n* = 3–4). *p* < 0.05 via ANOVA with Tukey’s post hoc analysis, **p < 0.001 and *p < 0.01. *p* values displayed in (D) and (F) are for the insulin+ cells. No significance was observed between the insulin– treatments. The scale bar is 10 μm and was added in postprocessing using ImageJ.

NCs can deliver cargo of interest to insulin-producing sBCs and human islets in vitro. After demonstrating that ENTPD3 antibody-coated Cy5-containing NCs are taken up by sBCs, we then assessed if NCs can deliver an additional cargo of interest to sBC clusters and human islets. To rapidly and effectively test β-cell-specific targeting, we utilized the β-cell toxin pentamidine (PTM) as a cargo. Empty (blank) or PTM-loaded NCs were left uncoated or coated with BSA (5 mg/mL), HA (0.2 mg/mL), ENTPD3 antibody (1μL/mL), or HA-Ex4 (0.2 mg/mL). Untreated and 1 μM free PTM (no NCs) treated sBC clusters were utilized as additional negative and positive controls. As there is no suitable assay to measure levels of the small molecule PTM, we were unable to measure the encapsulation efficiency of PTM in NCs; however, NCs were synthesized with 50 mg/mL PTM in the first water phase of the W/O/W synthesis method, which is ∼1000× more concentrated than the free pentamidine treatment in culture media at 1 μM. This allows for the functional measurement of PTM release by quantification of cell death over short periods of time where not all of the encapsulated cargo may be released. sBCs and human islets were stained to observe live (NucBlue) and dead cells (PI) (Figure 6A,B). To identify β-cells, human islets were also stained with Fluozin3 to identify the zinc-rich insulin granules in β-cells (Figure 6B), and in sBCs, we utilized the pINS·GFP reporter as previously described. As expected, the positive control 1 μM free PTM treatment killed the majority of cells in sBC clusters within 24 h (Figure 6C) while untreated samples exhibited low percentages of dead cells.^62^ Within 24 h of NC incubation with the sBC clusters, PTM-loaded ENTPD3-coated NCs had a significantly higher percentage of dead cells compared to the untreated control (*p* = 0.003, Figure 6A,C). A significant increase in cell death was observed with the PTM-loaded ENTPD3-coated NCs compared to the blank BSA-coated (*p* = 0.008), blank ENTPD3-coated control (*p* < 0.001), and BSA-coated PTM-loaded NCs (*p* = 0.01, Figure 6C). Significant cell death was also observed at 72 h when comparing the PTM-loaded ENTPD3-coated NCs to the untreated control (*p* = 0.05), blank BSA-coated (*p* = 0.008), blank ENTPD3-coated (*p* = 0.008), and BSA-coated PTM-loaded NCs (*p* = 0.008, Figure S5A,C,D). Except for the ENTPD3-coated PTM-loaded treatment, there was no significant change in sBC viability between the untreated sBCs and all other NC treatments (Figures 6C and S5A,C,D), strongly supporting that the NCs are nontoxic and that the amount of BKC on the NCs is below the cytotoxicity threshold. Further analysis was conducted where the number of GFP+ and GFP– dead cells were quantified as GFP correlates to insulin-positive cells. The free PTM treatment could not be quantified for GFP+ and GFP– sBCs due to too much loss of cellular structural integrity. For the dead sBC clusters, 76% at 24 h (Figure 6D) and 69% at 72 h were GFP positive (Figure S5D), supporting a high level of β-cell specificity and targeting in sBC. At 24 h, there was a significant increase in GFP-positive dead cells with PTM-loaded ENTPD3-coated NCs compared to the untreated (*p* = 0.008), blank BSA-coated (*p* = 0.004), blank ENTPD3-coated (*p* = 0.01), and BSA-coated PTM-loaded NCs (*p* = 0.008, Figure 6D). The increase in the percentage of GFP-positive dead cells was also significant at 72 h when comparing the PTM-loaded ENTPD3-coated NCs to the untreated (*p* = 0.05), blank BSA-coated (*p* = 0.008), blank ENTPD3-coated (*p* = 0.03), and BSA-coated PTM-loaded NCs (*p* = 0.008, Figure S5D). Collectively, this data indicates that BKC-PCL NCs are nontoxic and can be designed to target and deliver cargo to the cells of interest to sBC with high specificity and efficacy over 24–72 h.

The set of experiments described above was then repeated with human islets. Like the sBCs the PTM-loaded ENTPD3-coated and HA-Ex4-coated had a significantly higher percentage of cell death compared to their blank controls at both 24 and 72 h (*p* < 0.001, Figures 6E, and S5B,E). The HA-coated NCs had no significant change in the percentage of dead cells at both 24 and 72 h (Figures 6E and S5E). Furthermore, both the PTM-loaded ENTPD3 and HA-Ex4-coated NCs had significantly more insulin+ dead cells compared to their blank controls at 24 (*p* < 0.001 and *p* < 0.01) and 72 h (*p* < 0.001) (Figures 6F and S5F). Of interest, NCs targeted with either Ex4 or ENTPD3 showed similar levels of NC uptake, PTM-induced cell death, and β-cell specificity, indicating that ENTPD3 is a viable β-cell-specific targeting peptide. One limitation of interpreting islet cell viability using confocal microscopy is that diffusional limitations make it difficult to image the islet core; however, we have averaged cell death over three optical slices in each islet to represent cell death as accurately as possible. Additionally, while there is no suitable assay to measure the release profile of the small molecule PTM from NCs in vitro, functional analysis of PTM release via cell death over 72 h shows an increase in β-cell death with PTM-loaded NCs in both sBCs and human islets from 24 h to 72 h after treatment with NCs (Figure S5G,H), supporting sustained PTM release over 72 h in culture. Overall, these findings demonstrate that these NCs can encapsulate, selectively deliver, and release a cargo of interest to both sBCs and human pancreatic β-cells with a high level of specificity.

### HA-Ex4 NCs Enrich in Pancreatic Islet β-Cells In vivo NCs Enrich in Pancreatic Islet β-Cells In Vivo

**Figure 7:**
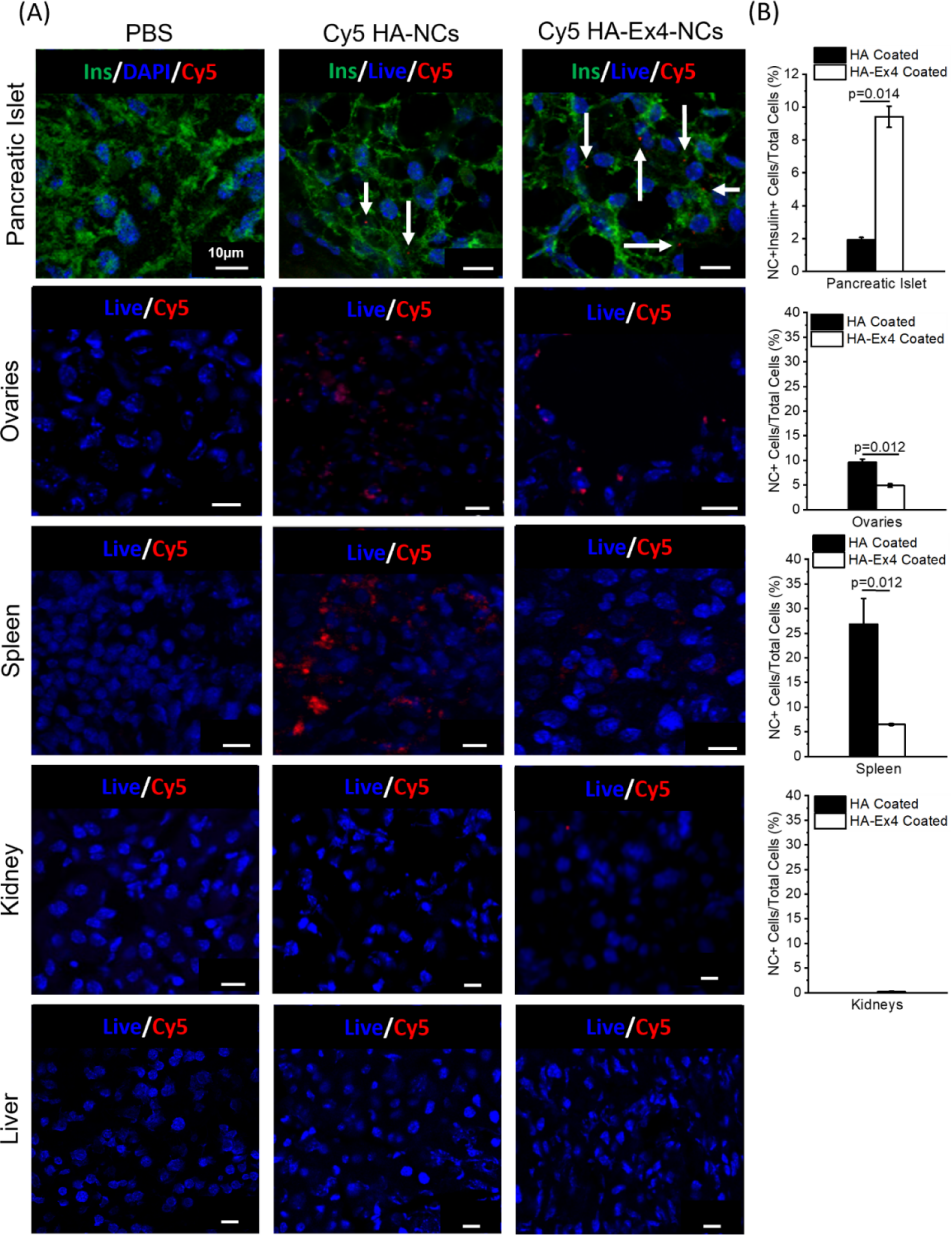
NCs can target pancreatic islet β-cells in vivo. (A) Representative confocal images of frozen pancreatic, ovary, spleen, kidney, and liver tissue sections from NOD-scid mice injected with either PBS only, Cy5-loaded HA-coated NCs, or Cy5-loaded HA-Ex4-loaded NCs. All tissues were stained for cell nuclei with DAPI (blue) and Cy5-loaded NCs (red). Pancreatic tissue was also stained for insulin (green). White arrows indicate Cy5 NCs. The scale bar is 10 μm. (B) Quantification of the percentage of NC+ cells per total cells in fixed tissues (*n* = 3). *p* < 0.05 via ANOVA with Tukey’s post hoc analysis. The scale bar is 10 μm and was added in postprocessing using ImageJ.

To test if Ex4-coated NCs could target pancreatic islets, Cy5-loaded HA-coated NCS, or Cy5-loaded HA-Ex4-coated NCs were injected into the tail veins of NOD-scid mice and then sacrificed 24 h post-injection. The staining of fixed pancreatic sections revealed an enrichment of NCs in pancreatic islets compared to the PBS control (Figure 7A, top row). Overall, we found ∼9.4% of all β-cells positive for NCs as indicated by Cy5 colocalization with insulin staining in mice treated with HA-Ex4-coated NCs, while only ∼1.9% of β-cells were positive for NCs in mice treated with HA-coated NCs, a ∼5 fold enrichment in NC uptake (*p* = 0.014, Figure 7B, top row). While off-target NC uptake was observed in the spleen and ovaries of animals treated with untargeted HA-only-coated NCs (Figure 7A,B, rows 2 and 3), off-target uptake was significantly reduced in β-cell-targeted HA-Ex4-coated NCs, further supporting β-cell-specific targeting of NCs in vivo. Additionally, no off-target NC uptake was observed in either liver or kidney sections 24 h after NC injection (Figure 7A,B, rows 4 and 5). Overall, the results of this study strongly support NC stability in the blood after tail vein injection and show proof of principle targeting of the mouse β-cell in vivo.

## Conclusion

In summary, in this study, we report the development and characterization of a stable, nontoxic NC drug delivery vehicle targeted to the human β-cell. We demonstrated that NCs coated with two different targeting peptides are taken up by human β-cells with high specificity in two complementary human islet models in vitro. Additionally, we demonstrated that NCs can deliver both hydrophilic and hydrophobic cargo to human β-cells. Finally, we have shown the proof of principle stability and β-cell targeting of NCs injected into the tail vein of NOD-Scid mice in vivo. Our unique NC design allows for the interchangeable coating of targeting peptides for future screening of targets with improved cell specificity. The ability to target and deliver therapeutics to human pancreatic β-cells opens avenues for improved therapies and treatments to help delay onset, prevent, or reverse T1D.

## Materials and Methods

### Materials

PCL *M*_w_ ∼ 14000 (cat. #440752), BKC (cat. #12060), coconut oil (cat. #C1758), acetone (cat. #270725), bovine serum albumin (BSA) (cat. #A2153), pentamidine isethionate salt (P0547), divinyl sulfone (DVS, cat. #V3700), tris(2-carboxyethyl)phosphine hydrochloride (TCEP, cat. #75259), and propidium iodide (cat. #P4170, Sigma) were all purchased from Sigma-Aldrich (Milwaukee, WI). 4,4-Difluoro-3a,4a-diaza-*s*-indacene fluorophore (BODIPY) (cat. #D3792), rhodamine-123 (Rh123) (cat. #cR302), NucBlue (cat. #R37605), 96 well plates (cat. #07-200721), Dulbecco’s modified Eagle’s medium (DMEM) (cat. #11-965-118), fetal bovine serum (FBS), penicillin, streptomycin, dialysis tubing (21-152-8), PBS tablets (cat. #BP2944100), FluoZin-3, AM, cell permeant (cat. #F24195), guinea pig IgG isotype (IgG) (cat. #NBP1970365), and nanosphere size standards (cat. #09-980-027) were purchased from Fisher Scientific (Pittsburgh, PA). Lacey Formvar/carbon-coated copper grid (cat. #01883-F, Ted Pella, Redding CA), hyaluronic acid (HA) (cat. #GLR001, R and D Systems, Minneapolis, MN), ethanol (cat. #111000200, Pharmco), insulin syringes (cat. #CMD2626, BRANDZIG), and cuvettes (part #759071D, Brandtech). Insulin ELISAs were purchased from ALPCO Diagnostics (cat. #80-INSHU-E10.1, Keewaydin Rockingham, NH). Ectonucleoside triphosphate diphosphohydrolase-3 (ENTPD3) antibodies, guinea pig antihuman NTPDase3 (hN3-2_c_), and mouse antihuman NTPDase3 (hN3-B3_s_) were purchased from Centre de Recherche du CHU de Québec – Université Laval CHUL (Québec, Canada). Dapi-Fluoromount-G (cat. #17984-24) was purchased from Electron Microscopy Sciences (Hatfield, PA). Insulin primary antibody (catalog no. ab63820) was purchased from Abcam (Cambridge, MA). Antirabbit secondary antibody Alexa Fluor 488 anti-IgG donkey (cat. #102649-730) was purchased from VWR (Radnor, PA).

### Nanocapsule Synthesis

PCL-NCs were synthesized via a water-in-oil (W/O) emulsion technique for encapsulating hydrophobic cargo using methods adapted from previously described protocols (Figure 1A).^15,49^ The oil phase consists of two solutions. The first is coconut oil (60 μL) dissolved in ethanol (750 μL), and the second is PCL (2 mg/mL) and BKC (20 wt/wt % PCL) dissolved in acetone (4.25 mL). Both solutions were combined and stirred briefly and then immediately poured into the aqueous phase, consisting of 10 mL of deionized water for 1–2 min with rigorous stirring. The ethanol and acetone were removed using a rotavapor (R-100, Buchi, New Castle, DE), leaving an NC suspension in DI water. NCs were washed twice by centrifugation at 20 000 rcf for 10 min and resuspending the NCs in fresh DI water. Various BKC concentrations, oil volumes, and stirring speeds were tested to identify the optimal synthesis conditions for the generation of stable NCs, as outlined in Table 1. W/O-synthesized BKC-PCL-NCs were loaded with 10 μL of either BODIPY or Cy5 dissolved in the ethanol solution, and the volume of cargo added was subtracted from the amount of ethanol added, allowing the total volume to remain at 750 μL. Coated W/O NCs were incubated either with BSA or HA for at least 30 min at room temperature and then washed via centrifugation as previously stated above. All parameters were tested using at least three separate NC batches.

A water-in-oil in water (W/O/W) double emulsion-solvent evaporation synthesis was used to encapsulate hydrophilic cargo in NCs, similar to a previously described method (Figure 2A).^63^ The primary emulsion (W_1_:O) phase consisted of hydrophilic cargo dissolved in DI water (W_1_) and coconut oil (O) in a ratio of 40:60. The DI water with the dissolved cargo was combined with the coconut oil and sonicated at 20% power for 3 min on ice. The W_1_:O emulsion was then combined with DI water (W) at a 1:10 ratio and sonicated at 20% power for 3 min (W/O/W). A solution containing PCL (2 mg/mL) and BKC (20 wt/wt % PCL) dissolved in 4.25 mL of acetone was added dropwise to the W/O/W emulsion under vigorous stirring for 2 min. The acetone was evaporated, and the NCs were washed as described above. The W_1_:O phase was tested at various ratios ranging from 25:75 to 50:50 and ratios of the (W_1_:O)/W_2_ emulsion were tested at volume ratios from 5:95 to 25:75 (Table 2). Coating the W/O/W NCs with either BSA or HA was the same as stated above. All parameters were tested in triplicate as the (W_1_:O) NCs stated above.

### Nanocapsule Characterization

#### NC Size, Polydispersity, and Zeta Potential

The mean particle diameter and PDI of the NCs were measured via dynamic light scattering (DLS), and the overall charge was measured via the zeta potential. Both DLS and zeta potential were measured with either a ZetaPALS (Brookhaven Instruments, Holtsville, NY) or a Malvern Zetasizer (Malvern Panalytical Ltd., UK). DLS measurements were taken of uncoated, BSA (5 mg/mL)-coated, and HA (0.2 mg/mL)-coated W/O NCs. Zeta potential measurements of W/O NCs were taken of blank, BODIPY-loaded, and Cy5-loaded samples either uncoated or coated at various concentrations ranging from 2–25 mg/mL of BSA and 0.2–5 mg/mL of HA (Figures 3B and S1A,B). Samples were diluted with DI water until the solution was clear, and 3 mL were placed in polystyrene spectrophotometry cuvettes for analysis. Each experimental condition was performed with three separate W/O batches.

The mean particle diameter and PDI of blank, Rh123 (0.06 mg/mL)-loaded, and pentamidine (PTM, 50 mg/mL)-loaded W/O/W NCs were also measured via DLS as stated above. Each experimental condition was performed with three separate W/O/W batches.

#### Electron Microscopy

DLS size measurements were confirmed via transmission electron microscopy (TEM) using an FEI Tecnai T12 transmission electron microscope (ThermoFisher Scientific, Waltham, MA) and scanning electron microscopy (SEM) using either a JEOL JSM 7000F field emission scanning electron microscope (Akishima, Japan) or a TESCAN S8252G RAMAN SEM/FIB (Brno, Czech Republic).

For TEM, 2 μL of NC suspension was mounted on a lacey Formvar/carbon-coated copper grid and air-dried before imaging. For SEM, samples were air-dried on carbon tape, gold-sputtered before imaging and imaged between 5 and 15 kV.

#### Confirmation of NC Coating

NCs were coated with 5 mg/mL of BSA or 0.2 mg/mL of HA as described in the NC Synthesis section. Their size, PDI, and zeta potential were then measured using the DLS instruments and techniques. Additionally, TEM assessed the NC size and provided visual evidence supporting NC coating. To further quantify the amount of HA coated onto the NCs and assess the stability of the NC coating after centrifugation. NCs were prepared as described above and then diluted down to an optical density (OD) of 20 ± 0.48 measured at a wavelength of 250 nm.

NCs were incubated with 0.2 or 2 mg/mL of TMR-HA for 30 min with stirring at 200 rpm at room temperature, after which 1 mL aliquots were centrifuged at 10 000 rpm for 10 min. The supernatant was decanted, and the remaining NCs were diluted with DI water up to 1 mL. This washing step was repeated 2 times to remove the excess TMR-HA that was not coated on the NCs. A 16-point TMR-HA standard curve was generated, which ranged in concentration from 0 to 0.5 mg/mL. Samples and standards, 200 μL each, were added to a black-walled, clear-bottom 96-well microplate in triplicate. The microplate was read with a Synergy H1 microplate reader (BioTek) with an excitation wavelength of 546 nm and an emission wavelength of 576 nm with a gain of 50. The fluorescence intensity of the standard curve was fit to the following equation in MATLAB: y = ae^bx + ce^dx. The coefficients generated from the line of best fit were used to solve for the concentration of the TMR-HA coated onto the NCs (*n* = 3).

#### Encapsulation Efficiency (EE) of NCs

BODIPY (W/O) or Rh123 (W/O/W) were encapsulated in BKC-NCs as described above. After synthesis and washing the NCs 2–3 times with DI water, the NC suspensions were dissolved in acetone, and 250 μL of samples were placed in triplicate in a 96-well plate. Standard curves were created by dissolving all components utilized for NC synthesis (excluding the DI water, acetone, and ethanol) in the same volume of acetone to emulate 100% encapsulation efficiency (EE) and diluted by factors of 1:2–1:1000 to generate a curve with a linear correlation between concentration and fluorescence. From the linear equation generated, all standard curves had a coefficient of determination (*R*^2^) of 0.99, supporting the interpolation of samples. The following equation was then used to calculate the EE:
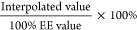
1

Fluorescence intensities of the samples were measured at 485/515 nm for BODIPY and 500/530 nm for Rh123 using a Synergy H1 microplate reader (Agilent Technologies, Santa Clara, CA) and analyzed via Gen5 3.11.19 software. Samples and standards were measured without a lid and set at a read height of 7 mm with a 100 ms delay, xenon flash, light source, and a gain of 100. All samples were read on the same plate at the same time as the standard curve samples to minimize the error between different plate readings.

#### Freeze-Drying NCs

Blank NCs at an OD of 20 were suspended in 5–10 mL of either DI water or 2.5 w/v, 5 w/v, or 10 w/v mannitol solutions. All solutions were stored at −80 °C overnight and then lyophilized for 24–48 h. Freeze-dried NCs were stored at room temperature and protected from light for one month. NCs were then resuspended in DI water and centrifuged at 20 000 rcf for 10 min. The mannitol solution was discarded and replaced with fresh DI water. NC size was analyzed by DLS and SEM (Figure S2A–C).

#### Assessment of NC Stability in vitro

The stability of both coated and uncoated NCs was tested in culture media and after being passed through a 29G 1 cm^3^ insulin syringe. Uncoated, BSA (5 mg/mL), and HA (0.2 mg/mL) W/O NCs were incubated in DMEM culture media with 10% FBS and 5% penicillin/streptomycin for 1 and 24 h both at room temperature and in an incubator set at 37 °C and 5% CO_2_. For the syringe transfer, 300 μL of uncoated, BSA (5 mg/mL), and HA (0.2 mg/mL)-coated W/O NCs were taken up and passed through a 30G insulin syringe needle. NC size and PDI were measured via DLS before and immediately after each treatment. Three separate W/O NC batches were tested (Figure S3A–C).

### In Vitro Cargo Release

BSA-coated NCs loaded with either BODIPY or Rh123 were placed in a dialysis bag (12 000–14 000 MWCO) in 415 mL of 1× phosphate-buffered saline (PBS). The beaker was sealed with parafilm to mitigate evaporation. The beaker was placed on a stir plate at 100 rpm, protected from light, and stored at 37 °C. 1 mL samples were withdrawn every 24 h for the first 15 days and then once every 5 days until Day 60. Experiments were performed at pH 7.4, 6, and 5, where the pH of each reservoir was checked weekly and adjusted with either 1 M HCl or NaOH as necessary. The fluorescence intensity of samples was measured at 485/515 nm for BODIPY and 500/530 nm for Rh123. Samples were measured in triplicate. The intensity at each time point was normalized by the 0-time point fluorescence intensity, and the normalized value was averaged across experimental replicates. The percent release of the fluorophore was calculated using the following equations:

Difference between fluorescence intensity at time 0 from each time point *I*_*N*_(*t*):
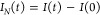
2

Percent release at each time point:
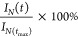
3

where, *I*(*t*) is the fluorescence intensity at a given time (*t*), *I*(0) is the intensity at time 0, and *t*_max_ is the time when the fluorescence intensity was at its maximal value. SEM imaging was utilized to visualize the degradation of NCs on Days 0, 28, and 60.

### Selectivity and Cytotoxicity of β-Cell Targeted NCs In Vitro

#### sBC Differentiation

Human pluripotent stem cells (hPSCs) containing a green fluorescence reporter were cultured and directly differentiated as previously described.^58,64,65^ Briefly, hPSC cultures were maintained on Geltrex-coated plates in mTeSR+ media (STEMCELL Technologies). To induce direct differentiation, hPSC cultures were transitioned into suspension-based bioreactor cultures for 72 h. Differentiating suspension cultures were monitored daily. sBCs emerged approximately on Day 13 of the differentiation process and were utilized for experiments in this study between Days 22 and 32.

#### sBC Characterization

Dynamic insulin secretion was measured using a BioRep Technologies perifusion machine (PERI4-115-1810-076) as previously described in Barra et. al 2024.^66^ Briefly, 50 sBC clusters/sample were run on a perifusion program consisting of a 30 min preincubation step with KRB buffer containing low (2.8 mM) glucose followed by 30 min of low glucose, 15 min of high glucose (20 mM), 15 min of high glucose + IBMX (50 μM), 15 min of low glucose, 5 min of KCl (30 mM), and 10 min of low glucose. Perifusion flow-through was collected in 96-well plates and stored at 4 °C overnight or at −20 °C if longer storage was needed for future analysis. Cell pellets were recovered, lysed with an acid/ethanol solution, and frozen overnight for the assessment of total insulin content.

Flow cytometry was performed on Day 23 sBC with standard methodologies previously described by Barra et al. in 2024, using an in-house Alexa 488 conjugated mouse monoclonal C-peptide antibody (Origene, #BM270).^66^

#### Human Cadaveric Islets

Human islets from cadaveric donors were obtained through the Integrated Islet Distribution Program (IIDP). Donor islet viability, purity, and donor characteristics, including age, sex, BMI, and race, are shown in Table 5.

**Table 5:** Human Cadaveric Islet Viability, Purity, and Donor Characteristics for All Donors Received from the IIDP

Donor ID	Viability (%)	Purity (%)	Age	Sex	BMI	Race
SAM N34075901	91	83	39	M	41.8	White
SAMN34130383	99	95	42	F	29.2	Hispanic/Latino
SAMN34411471	94	92	45	M	33.1	White
SAMN36704819	80	85	52	M	25.9	White
5AMN36705973	96	95	36	F	30.2	Black/African American
SAMN36823227	94	77	41	F	38.4	White
SAMN37638596	99	80	34	M	27.2	Native Hawaiian/Pacific Islander
SAMN38117428	95	95	47	M	28.1	White

#### Synthesis of Ex4 Conjugated to HA (Ha-Ex4)

The modification of HA with DVS was prepared as previously described with some modifications.^67^ The low molecular weight HA was dissolved in 0.1 M NaOH at 2% (w/v) (∼200 μmol of hydroxyl groups per mL). DVS was added to the HA solution on a magnetic stirrer at 1000 rpm at a molar ratio of 10× the hydroxyl groups of HA. The reaction was carried out for 10 min at room temperature and stopped by adjusting the pH to 5 using 1 M HCl. The solution was dialyzed (MWCO: 3.5 kDa) against deionized (DI) water (pH ∼ 5.3) every day with a new medium for 5 days and then freeze-dried. Cysteine-modified Ex4 was provided by Dr. David Hodson and Dr. Johannes Broichhagen and was synthesized as previously described.^68^ Before conjugating the modified HA to Ex4, the Ex4-Cys was also modified with a reducing agent, TCEP, at a molar ratio of 1:10 using DI at room temperature with stirring at 1000 rpm for 30 min and then freeze-dried.^69^ The modified dried forms of Ex4 and HA were redissolved in DI, and the pH of the solution was adjusted to 9 with 1 M HCl. The solution was incubated at 37 °C, 1000 rpm for 12 h in a nitrogen-purged flask on an oil bath. To block the unreacted acryloyl groups in HA-Ex4 conjugates, a 2 M excess of cysteine was added to the reaction solution. The pH of the reaction solution was adjusted to 9 again and incubated at 37 °C for another 12 h. Then, the reaction was stopped by reducing the pH to 7.0 with 1 N HCl. HA-Ex4 conjugates were purified by dialysis (MWCO: 3.5 kDa) against DI water (pH ∼ 7.2) every day with a new medium for 5 days and then freeze-dried.

#### Bioactivity of the HA-Ex4 Conjugate

To determine the bioactivity of HA-Ex4 conjugates, the enhancement of glucose-stimulated insulin secretion (GSIS) was measured in isolated islets. B6 mouse islets were cultured with 2 mM glucose in Krebs-Ringer buffer (recipe) with 0.1 wt % BSA (pH 7.4) and incubated for 1 h at 37 °C. The islets were then transferred into 500 μL of 2 mM, 11 mM, or 20 mM glucose in Krebs Ringer buffer with 0.1 wt % BSA, with or without HA-Ex4 (10 nM), and were then incubated with light shaking for 1 h at 37 °C. Five islets were analyzed per treatment, and all treatments per mouse were performed in duplicate. 350 μL of each treatment was transferred to microcentrifuge tubes and centrifuged at 5000 rpm for 5 min. The supernatants without the pellet were collected and stored in fresh microcentrifuge tubes. 250 μL of Krebs-Ringer buffer with 0.1 wt % BSA containing 2% Triton X-100 was added to the remaining 150 μL of each treatment and then transferred to microcentrifuge tubes (lysate). All supernatant and lysate samples were stored at −20 °C overnight. GSIS for each treatment was measured via enzyme-linked immunosorbent assay (ELISA) per the manufacturer’s instructions. Each microplate was read on a Synergy H1 microplate reader (BioTek) with an absorbance of 450 nm, and insulin concentration was calculated based on the standard curve per the manufacturer’s instructions.

#### NC Trafficking

GFP-expressing sBC were cultured with Cy5-loaded NCs, either uncoated or coated with an ENTPD3 antibody (1 μL/mL), for 24 h in mTeSR+ media. Human islets were cultured with Cy5-loaded NCs, uncoated or coated with BSA (5 mg/mL), HA (0.2 mg/mL), IgG (1 μL/mL), ENTPD3 (1 μL/mL), or HA-Ex4 (0.2 mg/mL) for 24 and 48 h. Human islets were stained with FluoZin-3, a zinc sensor that localizes to insulin granules, and NucBlue, a live cell dye. Clusters and human islets were imaged on a Leica STELLARIS 5 LIAchroic with a 40× water immersion objective, 405, 488, and 638 nm solid-state lasers, and 3 HyD spectral detectors. NC-positive (NC+) cells were quantified via manual counting in ImageJ, where any Cy5 signals surrounding individual nuclei stained by NucBlue were considered positive (NIH). NC-negative (NC−) cells were defined as cells that did not have NCs on the periphery or within the insulin or NucBlue stain. Insulin-positive cells were counted manually as determined by the presence of high fluorescence intensity staining of insulin granules; diffuse low-intensity FluoZin-3 staining in the cytoplasm was not considered insulin-positive.

#### Targeted Delivery

Pentamidine (PTM)-loaded NCs were synthesized using the W/O/W synthesis method described, above with the addition of 50 mg/mL (0.15M) of PTM dissolved in the water phase prior to synthesis. Blank and PTM-loaded NCs were coated either with an ENTPD3 antibody (1 μL/mL) or BSA (5 mg/mL) and added to sBC in culture medium for up to 168 h. sBC clusters were also treated with PTM only (1 μM) as a positive control. Clusters were stained with NucBlue (live) and propidium iodide (dead, 10 μL/mL). Clusters were imaged on a Leica STELLARIS 5 LIAchroic with a 40× water immersion objective, 405, 488, and 514 nm solid-state lasers, and 3 HyD spectral detectors. Live/dead cells were quantified via manual counting in ImageJ (NIH). 4–5 different sBC differentiations were used and 3–6 sBC clusters were imaged per treatment per experiment.

#### Flow Cytometry

After coculture with Cy5 NCs, wells with dispersed sBC were washed twice with 1× PBS to remove any NC not taken up by the cells. Cells were removed from the plate using TrypLE solution (Gibco 12604021) for 3 min and quenched with an equal volume of media. Cells were filtered through a cell strainer into FACS tubes, washed once with 1× PBS, and resuspended in 200 μL of FACS buffer before analysis on the Cytek Aurora 5L spectral cytometer. sBC cultured alone was used as a compensation control for pINS.GFP expression, and pure Cy5 NC were used as a compensation control for NC signal. 40 000 events were collected for each sample.

### NC Trafficking In Vivo

All animal studies were conducted at the University of Colorado Anschutz Medical Campus in accordance with the guidelines and relevant laws set by the University of Colorado and the National Institutes of Health for the care and use of laboratory animals. All procedures were approved by the University of Colorado Institutional Animal Care and Use Committee under protocol #00929. PBS, Cy5-loaded HA-coated NCs, or Cy5-loaded HA-Ex4-coated NCs were injected into the tail veins of 8-week-old female nonobese diabetic/severe combined immunodeficiency (NOD-scid) mice. These mice were sacrificed 24 h post-injection, and the pancreata were extracted. The pancreata were cryosectioned into 10 μm thick sections, placed on slides, and immunohistochemistry was performed. Frozen sections were rehydrated, air-dried, and then permeabilized with PBS containing 5% NDS and 0.25% Triton X-100 for 5 min. A blocking buffer consisting of 5% NDS and 0.1% Triton-X-100 was then applied to the sections for 5 min. Sections were washed with PBS, and then a PBS solution consisting of 5% NDS, 0.1% Trition-X-100, and an anti-insulin primary antibody (1:1000) was applied, and the tissues were incubated either at 4 °C overnight or at room temperature for 2 h. Sections were rinsed three times with PBS, and then a PBS solution containing 5% NDS and an antirabbit secondary antibody (1:500) was added to the sections and incubated at room temperature for 2 h, protected from light. DAPI fluoromount (cell nuclei stain) was added to the sections, and the slides were covered with a glass coverslip and sealed using nail polish. Pancreatic tissues were imaged on a Leica STELLARIS 5 LIAchroic with either a 40× water or 63× oil immersion objective, 405, 488, and 638 nm solid-state lasers, and 3 HyD spectral detectors. NC-positive and NC-negative dead cells were quantified via manual counting in ImageJ (NIH). Eight to ten images were taken per pancreatic tissue per treatment. Two to three mice were used per treatment.

### Statistical Analysis

Statistical analyses were performed using Origin software (OriginLab, Northampton, MA). Two-sample *t*-tests and one-way analysis of variance (ANOVA) with Tukey’s post hoc analysis were performed as indicated. A *p*-value of <0.05 was considered statistically significant.

## Supporting Information Available

Characterization of NC surface charge (Figure S1); stability after freeze-drying (Figure S2); stability under various physiological conditions (Figure S3); uptake of Cy5-loaded NCs in human islets after 48 h culture (Figure S4); analysis of cell death after 72 h of culture with PTM-loaded NCs (Figure S5) (PDF)

## Abbreviations

ANOVA: analysis of variance
BKC: benzalkonium chloride
BSA: bovine serum albumin
DI: deionized
DLS: dynamic light scattering
DMEM: Dulbecco’s modified Eagle’s medium
DYRK1A: dual specificity tyrosine-phosphorylation-regulated kinase 1A
EE: encapsulation efficiency
ENTPD3: ectonucleoside triphosphate diphosphohydrolase 3
Ex4: Exendin-4
FACS: fluorescence-activated cell sorting
GFP: green fluorescent protein
GLP-1: glucagon-like peptide 1
GLP-1R: glucagon-like peptide 1 receptor
HA: hyaluronic acid
HPSC: human pluripotent stem cell
NC: nanocapsule
NC+: nanocapsule positive
NC–: nanocapsule negative
PBS: phosphate-buffered saline
PCL: polycaprolactone
PDI: polydispersity index
PTM: pentamidine
Rh123: rhodamine 123
sBC: cell-derived β-like cells
SEM: scanning electron microscopy
T1D: type 1 diabetes
TEM: transmission electron microscopy
TMR: tetramethylrhodamine
W/O: water-in-oil
W/O/W: water-in-oil-in-water
