# Supplementary material for: Peptide-Coated Polycaprolactone-Benzalkonium Chloride Nanocapsules for Targeted Drug Delivery to the Pancreatic β-Cell
Source: ACS Appl Bio Mater. 2024 Sep 24;7(10):6451–66. doi: 10.1021/acsabm.4c00621 (PMC11498138; doi:10.1021/acsabm.4c00621)
Supplement: Supplementary file 1 — mt4c00621_si_001.pdf [file mt4c00621_si_001.pdf]

Peptide Coated Polycaprolactone-Benzalkonium Chloride Nanocapsules for Targeted Drug Delivery to the Pancreatic β-Cell

Jillian Collins[1], Jessie M. Barra[2], Keifer Holcomb[1], Andres Ocampo[1], Ashton Fremin[1], Austin Kratz[2], Jubril Akolade[1], Julianna K. Hays[1], Ali Shilleh[3], Amit Sela[1], David J. Hodson[3], Johannes Broichhagen[4], Holger A. Russ[2], *Nikki L. Farnsworth[1]

[1] Department of Chemical and Biological Engineering, Colorado School of Mines, Golden, Colorado
[2] Depart of Pharmacology and Therapeutics, Diabetes Institute, University of Florida, Gainesville, Florida
[3] Oxford Centre for Diabetes, Endocrinology and Metabolism (OCDEM), NIHR Oxford Biomedical Research Centre, Churchill Hospital, Radcliffe Department of Medicine, University of Oxford, Oxford, UK
[4] Leibniz-Forschungsinstitut für Molekulare Pharmakologie, Robert-Roessle-Str. 10, 13125 Berlin, Germany

*Corresponding Author Email: nfarnsworth@mines.edu

Figure S1

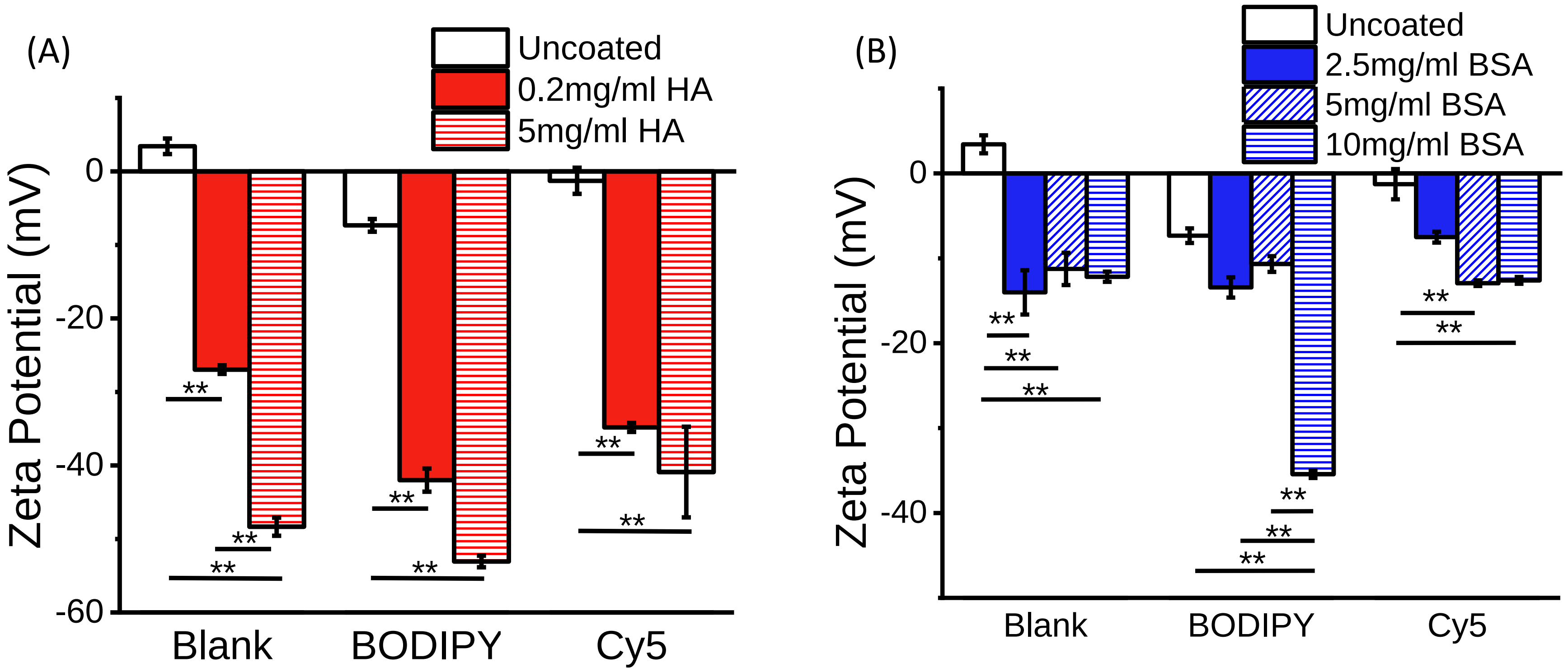

S1: Zeta potential measurements for blank, BODIPY, and Cy5 loaded W/O NCs coated with various concentrations of (A) HA and (B) BSA (n=3). $p<0.05$ was considered significant as determined via ANOVA with Tukey's post-hoc analysis, ** $p<0.0001$.

Figure S2

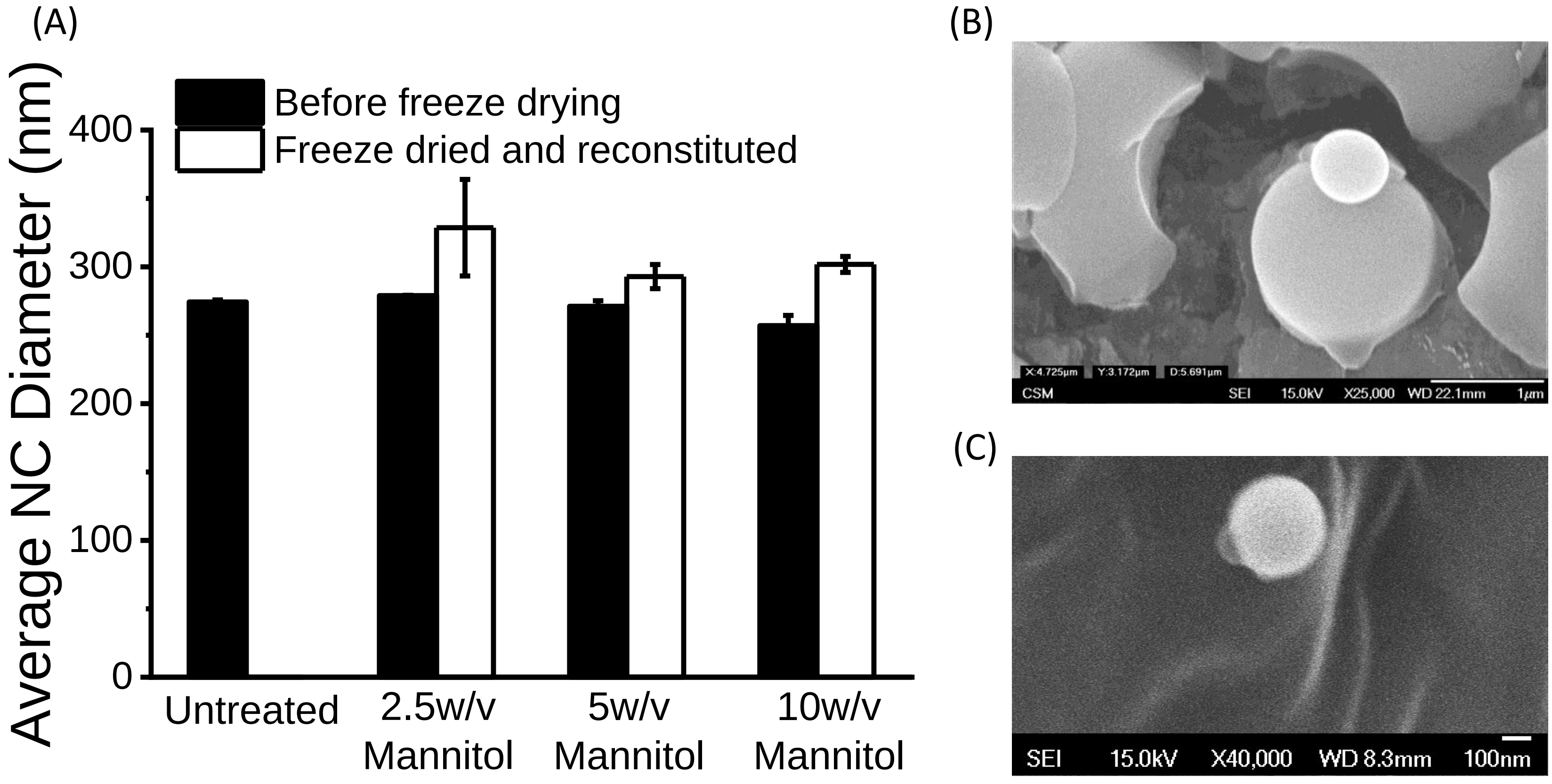

S2: (A) DLS measurements of average NC diameters before and after freeze drying with various w/v mannitol. SEM images of NCs (B) before and (C) after freeze drying and reconstituting. All PDIs were under 0.15 (n=2-3). Analyzed via ANOVA with Tukey's Post Hoc analysis, no conditions were statistically significant.

Figure S3

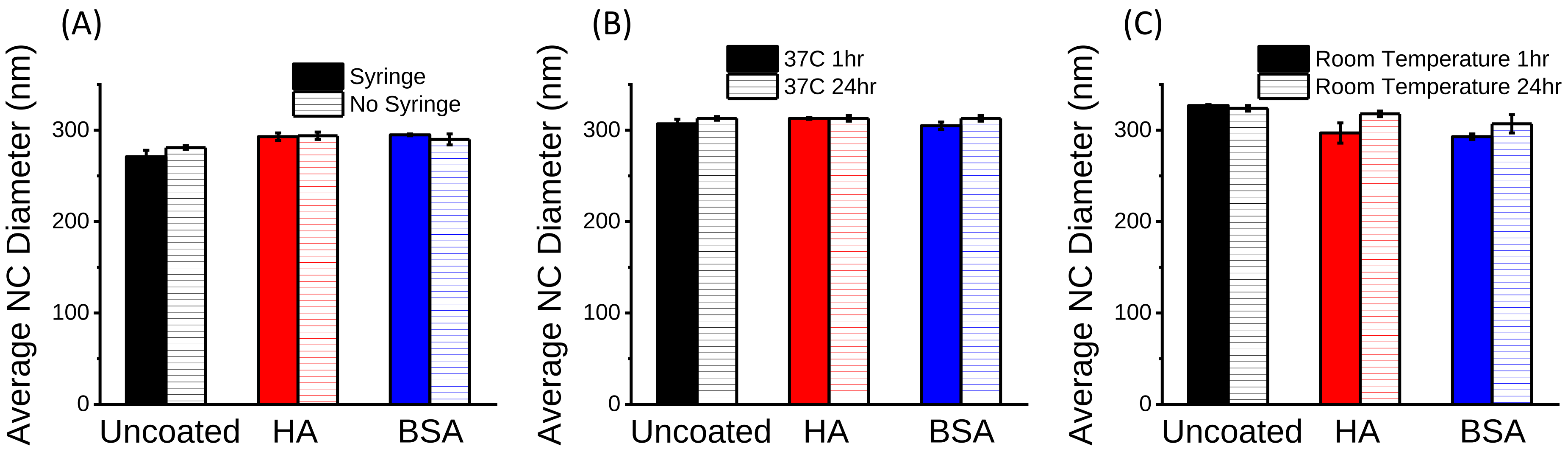

S3: DLS measurements of uncoated and coated W/O NCs (A) with and without being passed through a 29G 1cc insulin syringe, (B) after incubating for 24hr at 37C and (C) after incubating for 24hh room temperature all in in DMEM media. BSA and HA concentrations were 5mg/ml and 0.2mg/ml used to coat NCs. All PDIs were under 0.12 (n=3). Analyzed via ANOVA with Tukey Post Hoc analysis where no treatments were statistically significant.

Figure S4

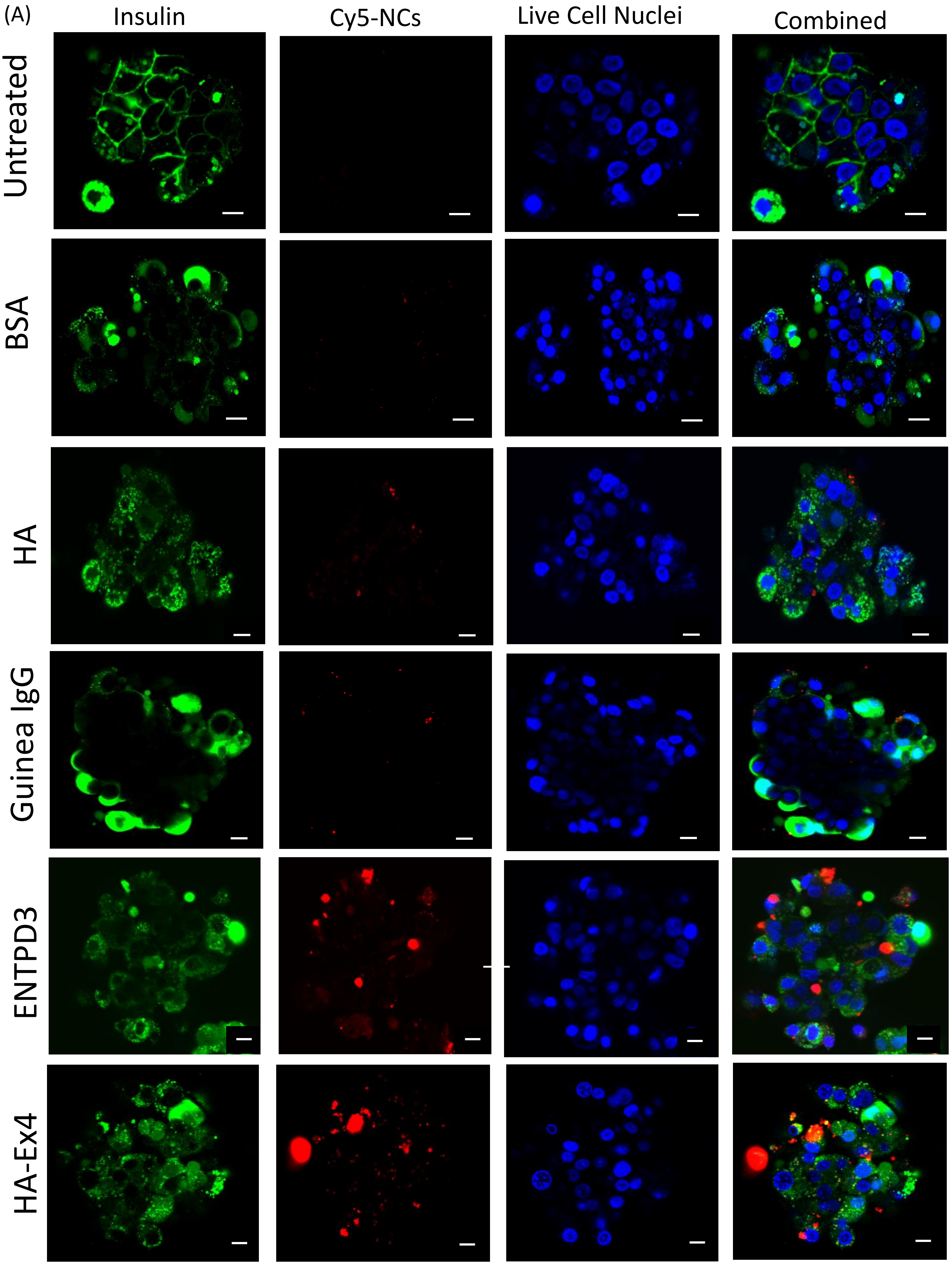

(A)
Insulin
Cy5-NCs
Live Cell Nuclei
Combined
Untreated
BSA
HA
Guinea IgG
ENTPD3
HA-Ex4

Figure S4

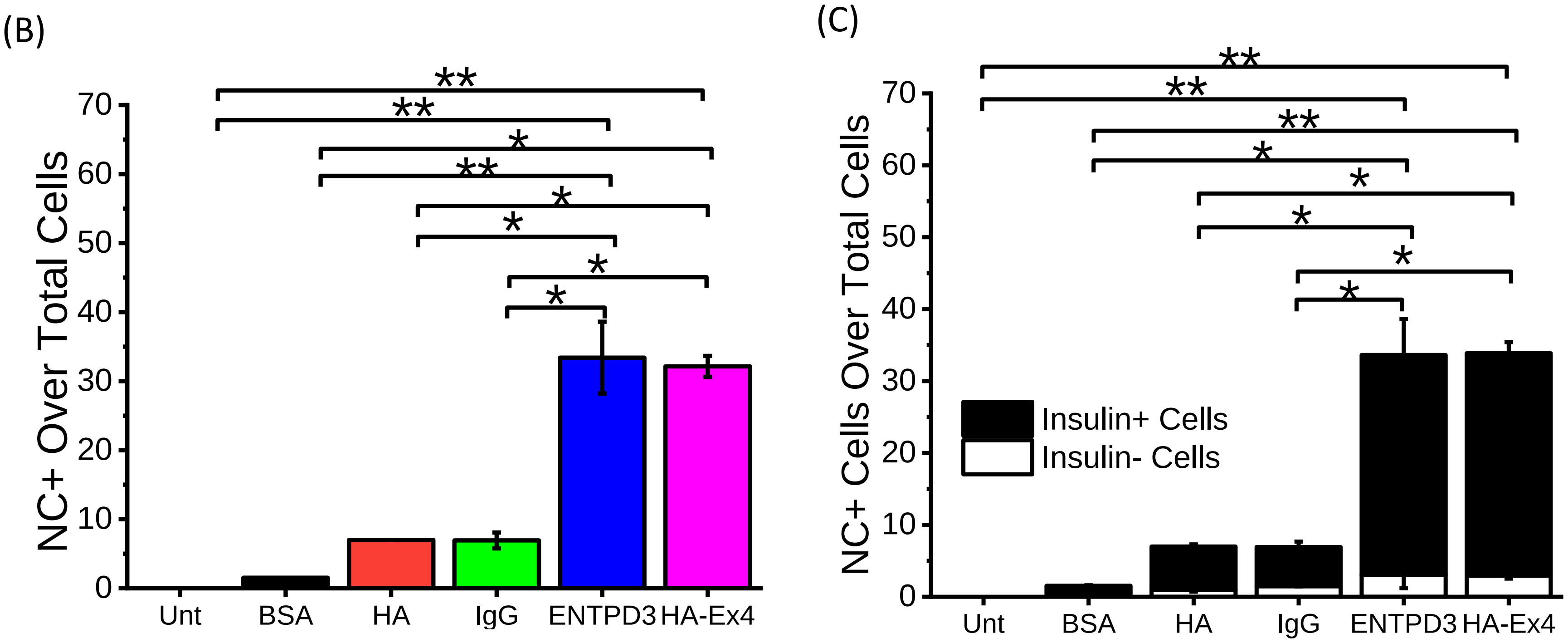

S4: (A) Representative confocal images showing insulin (green), live cell nuclei (blue) and Cy5 NCs (red) with various NC coatings in human islets . NCs were uncoated or coated with BSA (5mg/ml), HA (0.2mg/ml), guinea pig IgG (1µl/ml), guinea pig ENTPD3 antibody (1µl/ml), or HA-Ex4 (0.2mg/ml) and cultured with human islets for 48h (n=2-3). (B) Quantification of NC uptake with intact human islets for each NC treatment (n=2-3). (C) Quantification of insulin-NC+ and insulin+ NC+ cells with intact human islets for each NC treatment (n=2-3).p<0.001, *p<0.01, p<0.05 was considered significant as determined via ANOVA with Tukey's post-hoc analysis.**

Figure S5

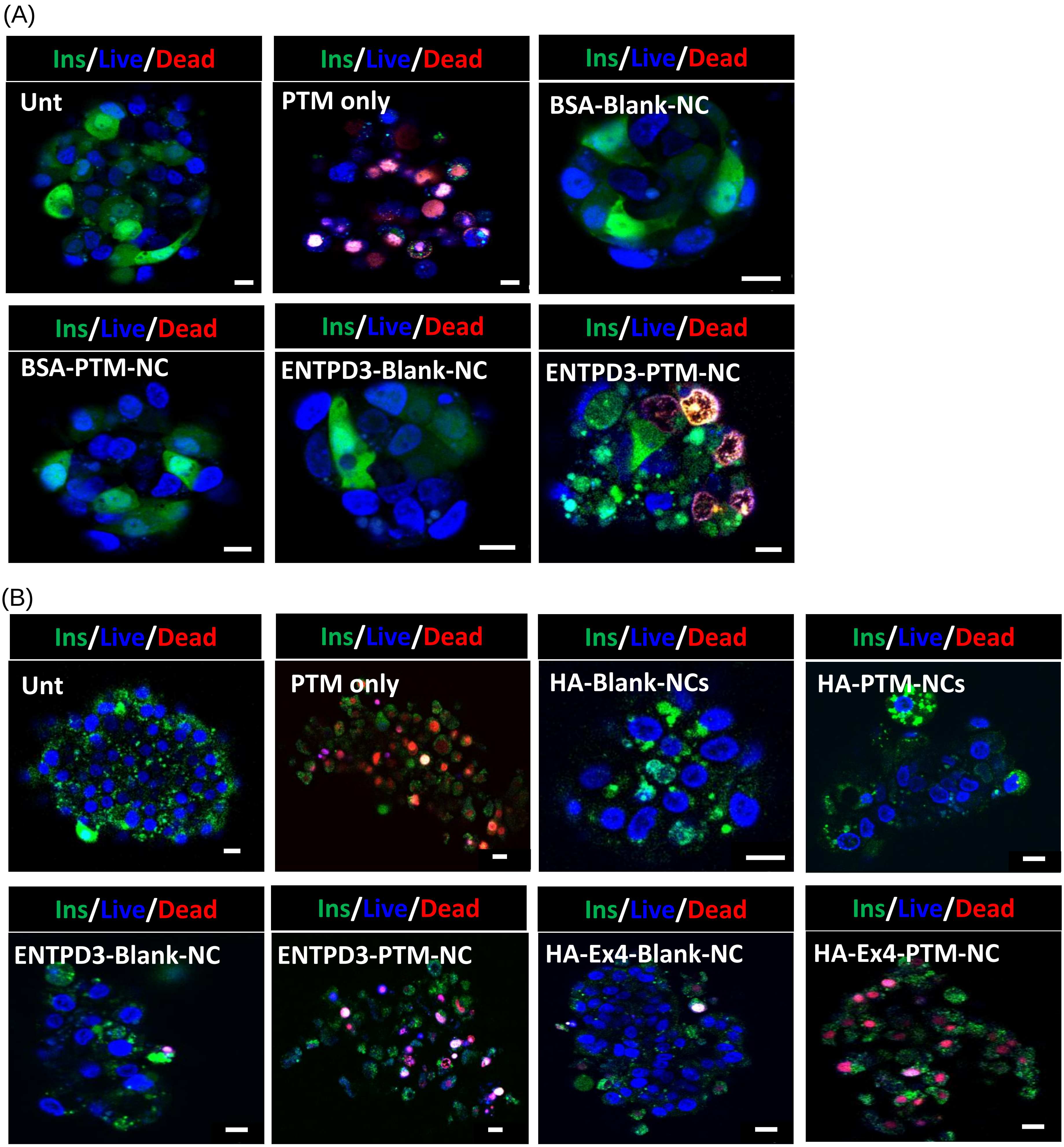
(A)
Ins/Live/Dead
Unt
Ins/Live/Dead
PTM only
Ins/Live/Dead
BSA-Blank-NC
Ins/Live/Dead
BSA-PTM-NC
Ins/Live/Dead
ENTPD3-Blank-NC
Ins/Live/Dead
ENTPD3-PTM-NC
(B)
Ins/Live/Dead
Unt
Ins/Live/Dead
PTM only
Ins/Live/Dead
HA-Blank-NCs
Ins/Live/Dead
HA-PTM-NCs
Ins/Live/Dead
ENTPD3-Blank-NC
Ins/Live/Dead
ENTPD3-PTM-NC
Ins/Live/Dead
HA-Ex4-Blank-NC
Ins/Live/Dead
HA-Ex4-PTM-NC

Figure S5

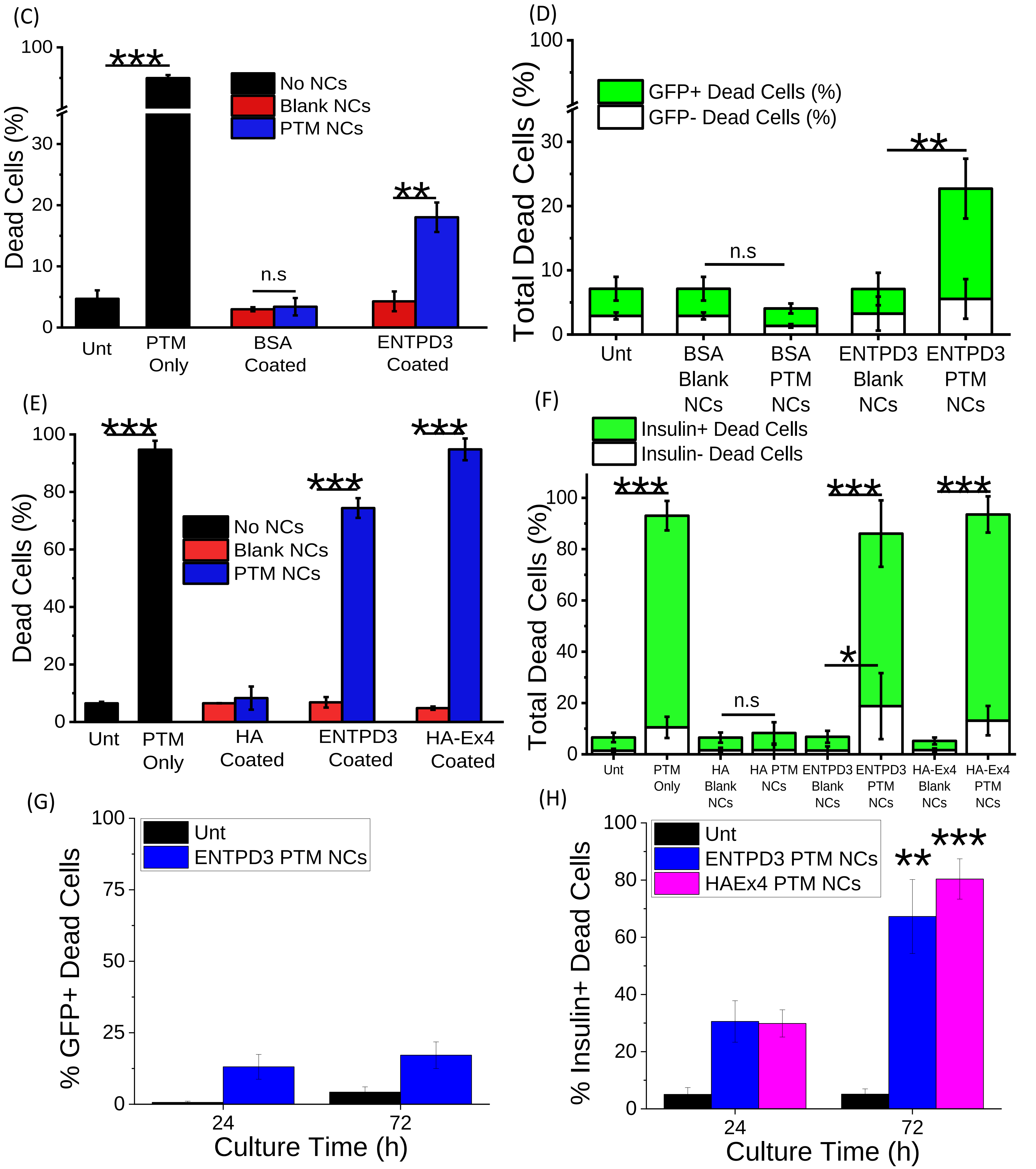

S5: (A) Representative confocal image of insulin producing sBCs (green), live cell nuclei (blue) and Cy5 NCs (red) with the various NC coatings at 72hr. Scale bar in all images is 10µm. (B) Representative confocal image of human islets stained for insulin (green), live cell nuclei (blue) and Cy5 NCs (red) with the various NC coatings at 72hr. Scale bar in all images is 10µm. Percentage of total dead sBCs for each NC treatment at 72hr (C) and a breakdown of insulin+ versus insulin- dead cells (D) (n=4-6). Percentage of total dead human islets for each NC treatment at 72hr (E) and a breakdown of insulin+ versus insulin- dead cells (F) (n=3-4). Percentage of GFP+ dead sBC (G) or insulin+ dead human β-cells (H) plotted with respect to time for each NC treatment shown using data from Figure 6 D and F and Figure S5D and F. ***p<0.001, **p<0.01, *p<0.05, p<0.05 was considered significant as determined via ANOVA with Tukey's post-hoc analysis. P-values displayed in (D) and (F) are for the insulin+ cells. No significance was observed between the insulin- treatments.
